# Landmarking the Brain for Geometric Morphometric Analysis: An Error Study

**DOI:** 10.1371/journal.pone.0086005

**Published:** 2014-01-28

**Authors:** Madeleine B. Chollet, Kristina Aldridge, Nicole Pangborn, Seth M. Weinberg, Valerie B. DeLeon

**Affiliations:** 1 Center for Functional Anatomy and Evolution, Johns Hopkins University School of Medicine, Baltimore, Maryland, United States of America; 2 Department of Pathology and Anatomical Sciences, University of Missouri School of Medicine, Columbia, Missouri, United States of America; 3 Center for Craniofacial and Dental Genetics, University of Pittsburgh School of Dental Medicine, Pittsburgh, Pennsylvania, United States of America; INSERM, France

## Abstract

Neuroanatomic phenotypes are often assessed using volumetric analysis. Although powerful and versatile, this approach is limited in that it is unable to quantify changes in shape, to describe how regions are interrelated, or to determine whether changes in size are global or local. Statistical shape analysis using coordinate data from biologically relevant landmarks is the preferred method for testing these aspects of phenotype. To date, approximately fifty landmarks have been used to study brain shape. Of the studies that have used landmark-based statistical shape analysis of the brain, most have not published protocols for landmark identification or the results of reliability studies on these landmarks. The primary aims of this study were two-fold: (1) to collaboratively develop detailed data collection protocols for a set of brain landmarks, and (2) to complete an intra- and inter-observer validation study of the set of landmarks. Detailed protocols were developed for 29 cortical and subcortical landmarks using a sample of 10 boys aged 12 years old. Average intra-observer error for the final set of landmarks was 1.9 mm with a range of 0.72 mm–5.6 mm. Average inter-observer error was 1.1 mm with a range of 0.40 mm–3.4 mm. This study successfully establishes landmark protocols with a minimal level of error that can be used by other researchers in the assessment of neuroanatomic phenotypes.

## Introduction

An examination of brain morphology, as defined by the size and shape of the brain as a whole and of individual structures within the brain, is one of the cornerstones of neuropsychiatric research and diagnosis. For example, age-related changes in brain morphology have been used to provide insight into the processes that underlie cognitive development throughout childhood and adolescence [1]–[2] and into those that underlie cognitive decline in senescence [3]. It has also been shown that brain morphology is altered in a variety of diseases (*e.g.*, type 2 diabetes [4]; major depressive disorder [5]; schizophrenia [6]; autism [7]) and thus has a practical role in clinical care and therapeutic research. Similarly, differences in brain morphology across species have been used to provide clues into human evolutionary history [8].

Traditionally, measures of size have been used to evaluate brain morphology. The use of these measures is based upon the idea that the size, and in particular the volume, of any given structure within the brain is determined by the functional requirements of that structure. Thus, a structure will be larger if it requires greater processing capacity and will be smaller if it requires less, such that form follows function [9]–[13]. However, there are notable exceptions to this tenet, including changes in size associated with certain pathological conditions. In these cases, a structure may undergo pathological enlargement rather than reduction in order to overcome functional deficits or as a result of the underlying disease process. For example, larger brain volume in children with autism has been attributed to alterations in the biochemistry governing apoptosis and synaptic density, abnormally enlarged neurons, and reduced synaptic density [14]. Measures of size have other limitations as well. As a univariate measure, volume does not provide the information necessary to determine whether an effect is global or local, whether and if so how regions within the brain are interrelated, or to quantify changes in shape that may be distinct even when size is not [15]–[18]. It is in these areas that an analysis of shape, rather than size, provides more extensive and appropriate information for study and comparison.

‘Shape’ is defined as the set of geometric properties of an object that are independent of position, size, and orientation [15]. A variety of techniques exist to characterize the shape of the brain (*e.g.*, outline analysis [19]–[20], deformation-based morphometry [21]–[23], surface-based morphometry for cortical folding patterns [24]–[25]), many of which are still evolving. The focus of this study is landmark-based statistical shape analysis. Landmark-based statistical shape analysis is a technique that has been used widely in the fields of anthropology, genetics, and evolutionary biology and has more recently emerged as a tool to assess brain shape. With regard to the brain, the strengths of landmark-based shape analysis are two-fold: (1) landmarks can be placed throughout the brain, creating a three-dimensional spatial map consisting of both cortical and subcortical structures; and (2) a variety of independent methods have been developed to analyze landmark data, permitting one to visualize and interpret data in a variety of ways [26]–[28]. The first point is critical to the utility of statistical shape analysis as a methodological technique in the evaluation of the brain, since the brain is believed to consist of a collection of networks running in series and parallel to achieve specific functions. Changes in the spatial arrangement of the components of these networks, and thus the shape of the brain either as a whole or regionally, likely reflect changes in functional capacity and execution [13], [17], [18], [29]–[32].

However, there are also limitations of landmark-based shape analysis. Landmarks can only be placed at locations that can be identified reliably on every individual under study. In some forms (or regions within a form), landmarks do not exist because there are not distinguishing features that reliably identify a particular point; thus, this technique may leave some regions underrepresented with potential overrepresentation of other regions. In addition, methods based on inferential statistics require that the number of variables does not exceed the degrees of freedom. Therefore, the number of landmarks that can be analyzed under these methods is limited by the sample size.

A more practical limitation of landmark-based statistical shape analysis of the brain is that, to date, none of the studies that have used this technique have published protocols for landmark identification and most have not published intra- or inter-observer error studies. Error studies were only available in the literature for three studies [33]–[35].

Of note, a variety of automated brain registration and cortical mapping programs have been validated that employ computer algorithms to delineate landmarks rather than rely upon manual landmark placement [36]–[42]. Automated methods for landmark placement have the benefit of removing inter-rater error and are especially useful for large sample sizes in which manual placement of landmarks would be grossly time consuming; however, as Pantazis et al. [43] has shown, automated methods may be less accurate in aligning occipital and frontal regions of the brain and these methods can be more susceptible to error when anatomical variation is high. Many automated methods are restricted to cortical landmarks, which limits their utility in the assessment of gross brain shape. Also, knowledge of different computational platforms is necessary to execute automated protocols and can be an impediment to novice researchers.

The purpose of the current study was to create a set of validated landmark protocols for the assessment of brain shape via landmark-based statistical shape analysis. Specifically, the primary aims were (1) to precisely and clearly define a set of landmarks that provide a biologically meaningful representation of brain shape, and (2) to evaluate the accuracy and repeatability of these landmarks, and by proxy, to optimize the landmark protocols in a multi-institutional intra- and inter-observer error study.

## Materials and Methods

This study was approved by the University of Iowa institutional review board and the Johns Hopkins institutional review board. Written informed consent was obtained from a parent or guardian for all children enrolled in the study. All participants signed an informed consent approved by the University of Iowa review board and were compensated for their participation in the study.

This study was completed using magnetic resonance images (MRIs) from ten healthy, right-handed, white males, age 12. Participants were originally recruited from the community by the University of Iowa. Exclusion criteria included braces and diagnosis of a major medical, neurologic, or psychiatric illness. This tightly constrained sample was chosen to limit variation among individuals.

Images were obtained using a 1.5-T Signa magnetic resonance scanner (General Electric, Milwaukee, Wisconsin) using a T1-weighted sequencing protocol. Voxel size was 1 mm×1 mm×1 mm. Post-acquisition processing was completed by technicians at the University of Iowa using the software BRAINS (Brain Research: Analysis of Images, Networks, and Systems) [44]–[47]. Post-acquisition processing consisted of brain extraction, in which the neural tissue was extracted from the surrounding skull and soft tissues of the face and scalp using an automated edge detection algorithm, followed by AC-PC alignment. The BRAINS software uses automated detection of the AC centroid, PC centroid, four ventricles, and mid-sagittal plane as defined by the interhemispheric fissure for spatial alignment. Details of linear alignment and post-processing are described elsewhere [44]–[48]. The use of the same verified automated processing technique across individuals minimized any innate error in the alignment process and thus would not be expected to have a significant impact on the reliability of manual landmark placement as was measured in this study.

As a baseline, landmarks were chosen according to (1) the frequency of their use in the literature and (2) the distribution of the landmark set with the goal of describing gross brain shape. In terms of frequency, landmarks were tabulated from the literature [17], [18], [33], [34], [49]–[54] (**Table 1**). “Common landmarks” were defined as those landmarks that have been used by at least three separate research teams in publication. “Uncommon landmarks” from the literature or novel landmarks were also included when the contributors agreed they were vital to the description of gross brain shape or of particular interest to the contributors. The final set of twenty-nine [29] landmarks (**Tables 2** **–** **3**) was determined by consensus among the contributors. Landmark data were collected for the left side of the brain only in order to limit the number of landmarks that needed to be collected, while still maintaining the diversity of landmarks being tested. The detailed landmark protocols established in this study are available in **Figure S1**.

**Table 1:** Brain landmarks tabulated from the literature.

Landmark	Aldridge	DeQuardo	Gharaibeh	Maudgil	Weinberg
amygdala	X				
anterior cingulate s/superior rostral s				X	
anterior commissure	X				
calcarine s./parieto-occipital s.				X	
caudate nucleus	X				
central s./lateral s.	X			X	X
central s.				X	
cerebellum - lateral pole					X
cerebellum - midsagittal inferior		X			X
cerebellum - midsagittal posterior					X
cerebellum - midsagittal superior		X	X		X
cerebral aqueduct/4th ventricle	X				
cingulate s.				X	
cingulate s./superior rostral s.				X	
corpus callosum - genu, anterior	X	X	X		X
corpus callosum - genu, posterior		X			
corpus callosum - midbody, inferior		X			
corpus callosum - midbody, sup		X	X		X
corpus callosum - splenium, ant		X	X		
corpus callosum - splenium, inf		X			
corpus callosum - splenium, post		X	X		X
fourth ventricle	X	X	X		
frontal pole	X		X		
inferior colliculus	X				
inferior frontal s./precentral s.	X			X	
lateral s./precentral s.				X	
lateral s./postcentral s.				X	
lateral s. – posterior termination	X				
lateral ventricle - anterior horn	X				
lateral ventricle - inferior horn	X				
lateral ventricle - posterior horn	X				
mammillary body	X				X
occipital pole	X				X
optic chiasm		X	X		
orbito-triangular s.					
parietooccipital s.				X	
pons - inferior	X	X	X		X
pons - superior	X	X	X		X
posterior commissure	X				
precentral s.				X	X
precentral s./superior frontal s.	X			X	
preoccipital notch				X	
superior colliculus	X	X	X		
thalamus	X				
temporal pole					X

Landmarks were tabulated from published studies where the primary methodology was landmark-based shape analysis of the brain in order to determine each landmark's frequency of use. Column headings indicate the source of the landmarks: (1) Aldridge [17], [49], [50]. (2) DeQuardo [51], [52]. (3) Gharaibeh [53]. (4) Maudgil [33], [54]. (5) Weinberg [34].

**Table 2:** Average intra-observer error and inter-observer error measured for landmarks.

		Intra-observer Error			Inter-observer Error		
#	LM	P1	P2	ΔP	P1	P2	ΔP
**1**	**Frontal pole**	1.5	-	-	0.88	-	-
**2**	**Occipital pole**	0.75	-	-	0.42	-	-
**3**	**Temporal pole**	1.2	-	-	0.71	-	-
**4**	**Central s./Lateral s.**	4.9	3.1	−1.8	3.1	1.9	−1.2
**5**	**Central s. – superior point**	4.0	3.2	−0.8	2.6	2.2	−0.4
**6**	**Pre-central s./Superior frontal s.**	4.6	4.9	+0.3	2.6	3.1	+0.5
**7**	**Pre-central s./Inferior frontal s.**	5.8	5.6	−0.2	3.3	3.4	+0.1
**8**	**Ascending ramus lateral s.**	3.3	2.7	−0.6	1.8	1.5	−0.3
**9**	**Horizontal ramus lateral s.**	2.9	2.8	−0.1	1.7	1.6	−0.1
**10**	**Superior temporal s.**	7.3	4.1	−3.2	4.1	2.4	−1.7
**11**	**Parieto-occipital s.**	2.9	2.8	−0.1	2.0	1.9	−0.1
**12**	**Cerebellum – lateral pole**	1.1	-	-	0.72	-	-
**13**	**Cerebellum – inferior pole**	1.9	1.0	−0.9	1.1	0.55	−0.55
**14**	**Cerebellum – posterior pole**	2.6	1.5	−1.1	1.5	0.84	−0.66
**15**	**Cerebellum – superior pole**	1.1	-	-	0.60	-	-
**16**	**Fourth ventricle**	0.95	-	-	0.54	-	-
**17**	**Amygdala**	1.9	1.7	−0.2	1.1	0.96	−0.14
**18**	**Caudate nucleus**	1.3	-	-	0.74	-	-
**19**	**Thalamus**	1.5	-	-	0.85	-	-
**20**	**Corpus callosum – genu**	0.89	-	-	0.50	-	-
**21**	**Corpus callosum – midbody**	1.5	-	-	0.94	-	-
**22**	**Corpus callosum – splenium**	0.78	-	-	0.44	-	-
**23**	**Anterior commissure**	0.76	-	-	0.44	-	-
**24**	**Pons - inferior**	0.93	-	-	0.53	-	-
**25**	**Pons - superior**	0.87	-	-	0.49	-	-
**26**	**Superior colliculus**	1.1	-	-	0.60	-	-
**27**	**Left ventricle - anterior horn**	0.72	-	-	0.40	-	-
**28**	**Left ventricle - inferior horn**	1.9	2.0	+0.1	1.2	1.2	0
**29**	**Left ventricle posterior horn**	4.1	1.7	−2.4	2.4	0.97	−1.4

In the landmark name, the backslash (/) indicates that the landmark is located at the intersection of the two sulci and s. is an abbreviation for sulcus. P1 is the imprecision (mm) for each landmark that was assessed in the first round of analysis. P2 is the imprecision (mm) for each landmark that was assessed in the second round of analysis using the modified protocols. The hyphen (-) indicates that the landmark was not reassessed in the second round of analysis because the error was less than 1.5 mm. ΔP is the difference between P2 and P1.

**Table 3:** Average intra-observer error by rater.

		Rater 1		Rater 2		Rater 3	
#	LM	P1	P2	P1	P2	P1	P2
**1**	**Frontal pole**	1.2	-	1.3	-	2.1	-
**2**	**Occipital pole**	0.93	-	0.61	-	0.72	-
**3**	**Temporal pole**	1.8	-	0.48	-	1.3	-
**4**	**Central s./Lateral s.**	6.6	4.8	0.74	0.99	7.4	3.6
**5**	**Central s. – superior point**	7.3	6.8	2.7	1.1	2.1	1.8
**6**	**Pre-central s./Superior frontal s.**	6.3	9.0	4.6	3.5	3.1	2.3
**7**	**Pre-central s./Inferior frontal s.**	5.2	9.2	4.0	3.6	8.1	3.9
**8**	**Ascending ramus lateral s.**	3.3	3.6	2.4	2.4	4.2	2.0
**9**	**Horizontal ramus lateral s.**	4.3	4.0	1.5	1.8	2.8	2.5
**10**	**Superior temporal s.**	8.3	5.3	5.1	2.4	8.3	4.7
**11**	**Parieto-occipital s.**	5.9	5.8	1.3	0.87	1.4	1.6
**12**	**Cerebellum – lateral pole**	2.1	-	0.48	-	0.74	-
**13**	**Cerebellum – inferior pole**	2.6	1.1	1.3	0.98	1.9	0.96
**14**	**Cerebellum – posterior pole**	2.2	1.3	1.7	1.6	3.8	1.7
**15**	**Cerebellum – superior pole**	0.96	-	0.76	-	1.4	-
**16**	**Fourth ventricle**	0.92	-	0.64	-	1.3	-
**17**	**Amygdala**	2.3	2.1	1.5	1.4	1.9	1.8
**18**	**Caudate nucleus**	1.5	-	0.92	-	1.5	-
**19**	**Thalamus**	1.8	-	1.1	-	1.6	-
**20**	**Corpus callosum – genu**	1.1	-	0.59	-	0.96	-
**21**	**Corpus callosum – midbody**	2.7	-	0.73	-	0.99	-
**22**	**Corpus callosum – splenium**	0.95	-	0.55	-	0.85	-
**23**	**Anterior commissure**	1.1	-	0.45	-	0.74	-
**24**	**Pons - inferior**	1.2	-	0.57	-	0.98	-
**25**	**Pons - superior**	1.2	-	0.63	-	0.81	-
**26**	**Superior colliculus**	1.4	-	0.68	-	1.1	-
**27**	**Left ventricle - anterior horn**	0.92	-	0.69	-	0.54	-
**28**	**Left ventricle - inferior horn**	3.0	2.6	0.73	1.1	2.0	2.3
**29**	**Left ventricle posterior horn**	5.8	1.2	4.0	1.5	2.5	2.4

In the landmark name, the backslash (/) indicates that the landmark is located at the intersection of the two sulci. P1 is the imprecision (mm) for each landmark in the first round of analysis. P2 is the imprecision (mm) for each landmark in the second round of analysis using the modified protocols. The hyphen (-) indicates that the landmark was not reassessed in the second round of analysis.

Three of the contributors participated in data collection for the error study. Familiarity with neuroanatomy and brain landmarks varied among the raters from a complete novice brain landmarker (Rater 3) to an advanced brain landmarker (Rater 1). This spectrum of expertise was intentional, because it captured the range of likely users of the landmark definitions and of the protocols under study.

Landmark coordinate data were collected from three-dimensional reconstructions of brain imaging data in eTDIPS (http://www.cc.nih.gov/cip/software/etdips/) [55]–[56]. Raters initially conducted 3 trials per individual with each trial separated by at least 24 hours (10 individuals×3 raters×3 trials×29 landmarks). A rater was not allowed to return to any trial once it had been completed. Landmark precision was calculated using the following formula:
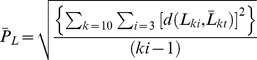
where 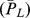 = estimate of placement error for any given landmark, 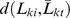 = distance from replicate landmark location to mean landmark location, *k* = individual, *i* = trial. Landmark precision for a given rater was thus calculated as the square root of the squared distance of a landmark trial to the mean position of that landmark for an individual subject, summed across individuals and divided by the total number of measurements minus one. Similarly, intra-rater error was calculated as the deviation of each landmark trial from the average landmark position *for that rater*, summed across trials, across raters, and across individuals, and divided by the total number of measurements minus one. Inter-rater error was calculated as the deviation of the average landmark position for each rater from the average position *across raters*, summed across raters and across individuals, and divided by the total number of measurements minus one.

Any landmark with average intra-observer error greater than 1.5 mm (when rounded to two significant digits) was reassessed in a second round of error analysis. For the second round, the protocols of these “problem landmarks” were modified to provide additional clarity. The same three raters completed three additional trials per individual using the new (modified) protocols with each trial separated by at least 24 hours (15 landmarks×10 individuals×3 trials×3 raters). Intra-observer and inter-observer errors were calculated as described above. Raw coordinate data for all trials are available in **Table S1**.

## Results

**Figure 1:**
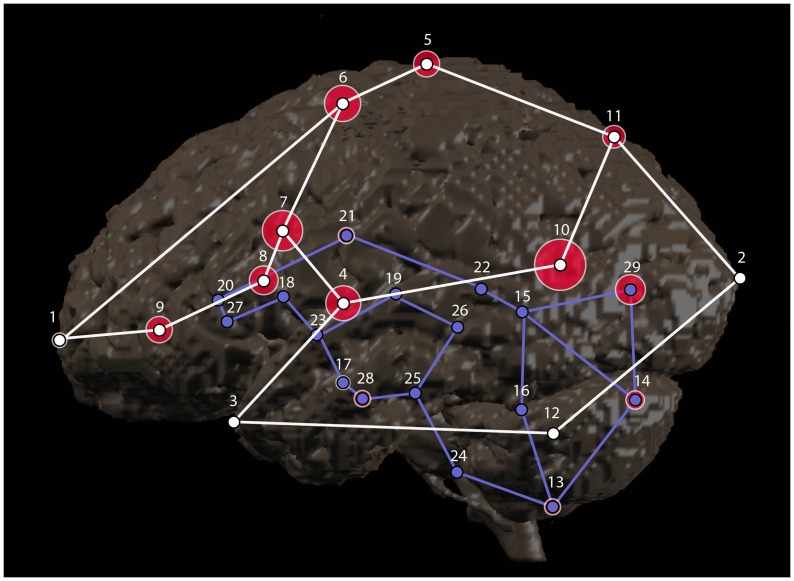
Landmarks and the associated error analyzed in this study. Left lateral view of a 3D reconstruction of the brain (anterior is to the left). Projected positions of landmarks are shown with numbers corresponding to Table 2. Cortical surface landmarks are white with white wireframe; subcortical landmarks are purple with purple wireframe. The size of the pink ellipses around each landmark indicate the magnitude of average precision (error) at anatomic scale. Landmarks for which no ellipse is visible had average error less than the 1.5 mm radius of the landmark marker. Note that the greatest magnitudes of error were associated with cortical surface landmarks.

Twenty-nine cortical and subcortical brain landmarks (**Table 1** **;** **Figure 1**) were assessed in this study (see Materials and Methods for selection criteria and definition of protocols). Using the initial set of protocols devised by the study team, average intra-observer error ranged from 0.72 mm (left ventricle anterior horn) – 7.3 mm (superior temporal sulcus) (**Table 2** **;** **Figures 1** **–** **2**). Sixteen of the 29 landmarks (55%) had an average intra-observer error of less than or equal to 1.5 mm, six (21%) had an average intra-observer error of 1.6–3.0 mm, and seven (24%) had an average intra-observer error greater than 3 mm. Inter-observer error ranged from 0.40 mm (left ventricle anterior horn) – 4.1 mm (superior temporal sulcus) (**Table 2** **;** **Figures 1** **,** **3**). Twenty of the 29 landmarks (69%) had an average inter-observer error of less than or equal to 1.5 mm, six (21%) had an average inter-observer error of 1.6–3.0 mm, and three (10%) had an average inter-observer error greater than 3 mm. The range of error and the landmarks associated with the greatest error were consistent among raters (**Table 3**). The range of error for each rater for the initial set of protocols was: Rater 1 = 0.92–8.30 mm, Rater 2 = 0.45–5.1 mm, and Rater 3 = 0.54–8.3 mm.

**Figure 2:**
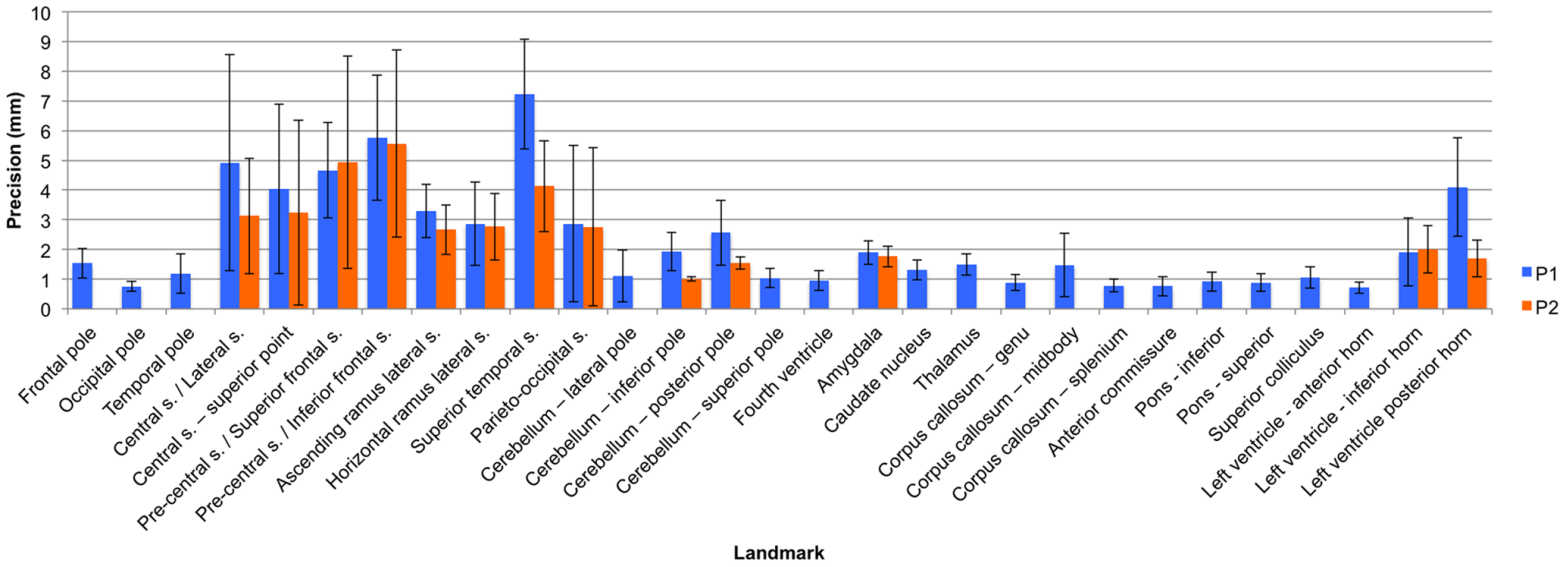
Histogram of the intra-observer precision of each landmark. This histogram indicates the level of intra-observer precision associated with each landmark using the original (P1) and modified (P2) protocols. The error bar is equal to one standard deviation above and below the mean. Landmark numbers correspond with the landmark numbers in Table 2.

**Figure 3:**
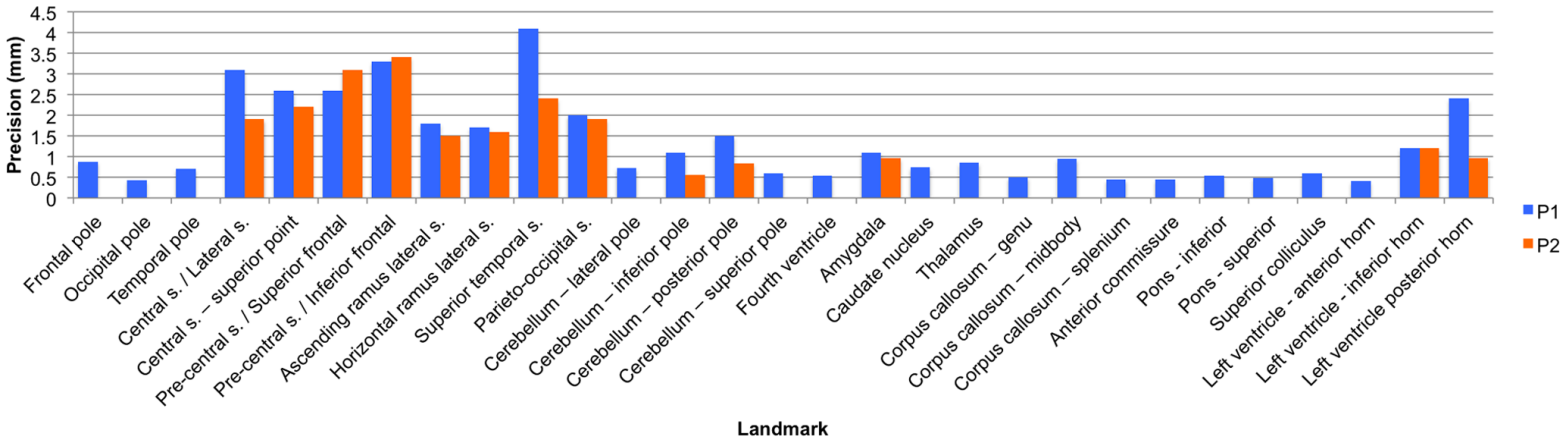
Histogram of the inter-observer precision of each landmark. This histogram indicates the level of inter-observer precision associated with each landmark using the original (P1) and modified (P2) protocols. Landmark numbers correspond with the landmark numbers in Table 2.

Midsagittal landmarks tended to have the least amount of error, while cortical and ventricular landmarks had the greatest error. Visual inspection of landmark placement revealed that most of the error for cortical landmarks was due to occasional misidentification of the central and pre-central sulci and the opercular and triangular sulci. Error in the inferior and posterior horns of the ventricles seemed to reflect the amount of CSF in the ventricles. Larger ventricles, filled with less radio-opaque CSF, tended to be associated with less landmark error, because the boundaries were more clearly defined. The terminations of narrower, more tapered ventricles were much less distinct. Moderate error in cerebellar landmarks resulted from confusion about whether landmarks should be placed on the vermis or lobar tissue. These issues were addressed by clarifying protocols and by providing additional information on how to identify cortical sulci.

The thirteen (13) landmarks that had an average intra-observer error of greater than 1.5 mm were re-collected using modified protocols with greater specificity in landmark definition. Average intra-observer error of the new data ranged from 1.0 mm (cerebellum – inferior pole) – 5.6 mm (pre-central s./inferior frontal s. intersection) (**Table 2**). Two (15%) of the 13 landmarks had an average intra-observer error of less than or equal to 1.5 mm, six (46%) had an average intra-observer error of 1.6–3.0 mm, and five (38%) had an average intra-observer error greater than 3 mm. Inter-observer error ranged from 0.55 mm (cerebellum – inferior pole) – 3.4 mm (pre-central s./inferior frontal s. intersection). Six of the 13 landmarks (46%) had an average inter-observer error of less than or equal to 1.5 mm, five (38%) had an average inter-observer error of 1.6–3.0 mm, and two (15%) had an average inter-observer error greater than 3 mm. Average intra-observer error decreased for eleven out of the thirteen landmarks using the modified protocols. Intra-observer error increased by 0.3 mm for the intersection of the precentral sulcus with the superior frontal sulcus and increased by 0.1 mm for the inferior horn of the lateral ventricle. Inter-observer error decreased for every landmark but two – pre-central s./inferior frontal s. intersection (+0.1 mm) and pre-central s./superior frontal s. intersection (+0.5 mm) – using the modified protocols.

The original landmark protocols, rather than the modified protocols, for the precentral s./superior frontal s. and the lateral ventricle-inferior horn were thus included in the final landmark protocol set. All other modified protocols were included in the final landmark set. Overall, the landmarks with the greatest intra- and inter-observer error measures were located at the superior temporal sulcus and at the intersection of the pre-central sulcus with the superior frontal sulcus and the inferior frontal sulcus. The range of error and the landmarks associated with the greatest error were consistent between Raters 2 and 3, but Rater 1, who was also the most experienced rater, had consistently greater error for cortical landmarks (**Table 3**). The range of error for the final set of landmarks for each rater was: Rater 1 = 0.92–9.2 mm, Rater 2 = 0.45–4.6 mm, and Rater 3 = 0.54–4.7 mm.

## Discussion

This study established protocols for collecting three-dimensional coordinate data for 29 cortical and subcortical brain landmarks. The average intra-observer error was 1.9 mm with a range of 0.72 mm–5.6 mm, and the average inter-observer error was 1.1 mm with a range of 0.4 mm–3.4 mm. Error was particularly high for landmarks located at the intersection of the pre-central sulcus and inferior frontal sulcus, the intersection of the pre-central sulcus and inferior frontal sulcus, and the superior temporal sulcus. The increased level of error for these landmarks should be taken into account both when deciding which landmarks to collect for a given study and, if these landmarks are employed, during data analysis. Notably, error can be further minimized by completing multiple landmarking trials, calculating the average landmark coordinates, and then conducting statistical analyses using these averaged data [57].

Intra- and inter-observer error was consistent with, and often better than, previous studies using landmark-based statistical shape analysis of the brain. Maudgil et al. [33] reported a mean intra-rater precision of 3.7 mm and inter-rater precision of 6.0 mm for 12 cortical landmarks. Aldridge [35] reported a mean intra-observer precision of 2.17 mm, with error ranging from 0.61–7.51 mm. Weinberg et al. [34] reported intra-class correlation coefficients of 0.86–1.0, which are not directly comparable to results in this study. Although the error was consistent with what has been reported in the literature, it remains unclear what the functional implications of this level of error are. The functional boundaries of cortical regions are diffuse, and even well circumscribed anatomical structures within the brain such as the thalamus or caudate nucleus are not limited to a single function. It is thus possible that an error of even 1.5 mm could impact a study's results.

One of the overarching goals of this study was to create a resource that could be used by both students and researchers who are new to the field of brain landmarking and advanced landmarkers as a reference source. In alignment with this goal, the aim was to create protocols that could be collected using existing morphometric software packages and not rely upon cortical parcellation or functional brain mapping before landmark placement. This meant creating a set of protocols that went beyond a single line definition of the landmark and included step-by-step specifications with associated images. When assessing the validity of these protocols, Rater 3 was chosen because she only had cursory knowledge of brain structure and had never before landmarked the brain. The only guidance she was given before execution of the project was a single 2-hour review session on the location of cortical and subcortical structures. As the ranges of intra-rater error indicate (Rater 1 = 1.07–9.19 mm, Rater 2 = 0.45–5.11 mm, and Rater 3 = 0.54–8.34 mm), researchers who have limited knowledge of brain landmarks can successfully follow these protocols.

The primary limitation of this study is that the sample size was limited to ten subjects. A sample size of ten was chosen to provide a sufficient level of evaluation while also making it possible to complete each landmarking trial in a single sitting. In addition, it is notable that this study was completed on a set of 12-year-old Caucasian males. It is possible that the protocols established in this study are not as accurate for individuals of a different age due to subtle changes in brain morphology with aging, but unlikely considering that most landmarks were defined by stable boundaries such as the intersection of two sulci or the centroid of a subcortical structure.

In summary, this study established detailed protocols with a minimal level of error for a set of twenty-nine subcortical and cortical landmarks. Future work includes the definition of additional landmarks relevant to hypotheses about brain shape, establishment and testing of protocols for these landmarks, and continued refinement of existing protocols in response to documented anatomical variation at landmark sites.

## Supporting Information

Figure S1 **Landmark guide.** The landmark guide includes detailed step-by-step directions for the location of the 29 landmarks assessed in this study.
(PDF)

Table S1 **Raw landmark coordinate data.** This table includes the raw coordinate data for all of the landmark trials for all three raters.
(XLSX)
