# Supplementary material for: Landmarking the Brain for Geometric Morphometric Analysis: An Error Study
Source: PLoS One. 2014 Jan 28;9(1):e86005. doi: 10.1371/journal.pone.0086005 (PMC3904856; doi:10.1371/journal.pone.0086005)
Supplement: Figure S1 — Landmark guide. The landmark guide includes detailed step-by-step directions for the location of the 29 landmarks assessed in this study. (PDF) [file pone.0086005.s001.pdf]

Brain Landmark Protocols

I. Cortical Landmarks
frontal pole (fp)
occipital pole (op)
temporal pole (tp)
central s. / lateral s. (cl)
central s. – most superior point (cs)
precentral s. / superior frontal s. (prsf)
precentral s. / inferior frontal s. (prif)
opercular s. (ope)
triangular s. (tri)
superior temporal s. – posterior termination (stp)
parietooccipital s. – most superior point (pos)

II. Cerebellar Landmarks
cerebellum – lateral pole (cbl)
cerebellum – midsagittal inferior (cbi)
cerebellum – midsagittal posterior (cbp)
cerebellum – midsagittal superior (cbs)
fourth ventricle – posterior-most point (fv)

III. Subcortical Landmarks
amygdala (centroid) (amyg)
caudate nucleus (centroid) (cn)
thalamus (centroid) (thal)
corpus callosum – genu, anterior (ccga)
corpus callosum – midpoint, superior (ccms)
corpus callosum – splenium, posterior (ccsp)
anterior commissure (midline) (ac)
pons – inferior (ponsi)
pons – superior (ponss)
superior colliculus (scol)

IV. Ventricular Landmarks
lateral ventricle – anterior horn (lva)
lateral ventricle – inferior horn (lvi)
lateral ventricle – posterior horn (lvp)

Frontal Pole (FP)

Definition: The most anterior point of the frontal lobe of the cerebrum.

Location: Use the coronal view to identify the most anterior slice of the frontal lobe. *(Note: Look for the anterior-most slice where neural tissue is visible. Be cognizant of residual dura.*) If there is more than one pixel of visible neural tissue, place the landmark in the center of the neural tissue mass. Confirm landmark placement using the axial and sagittal slices.

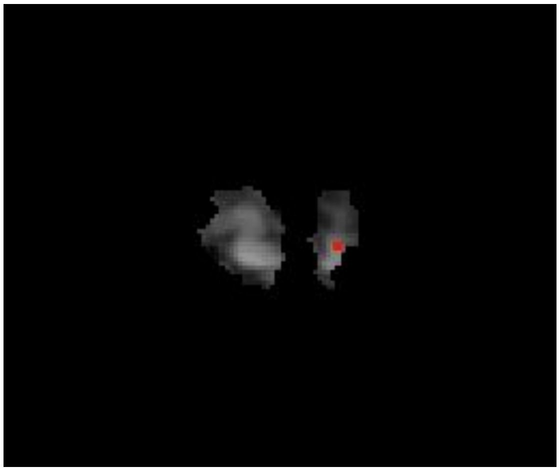

CORONAL

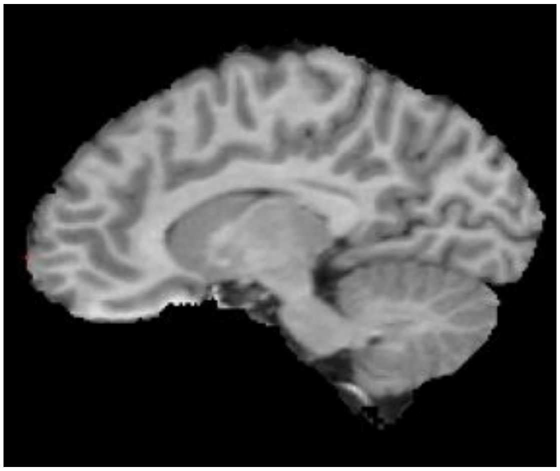

SAGITTAL

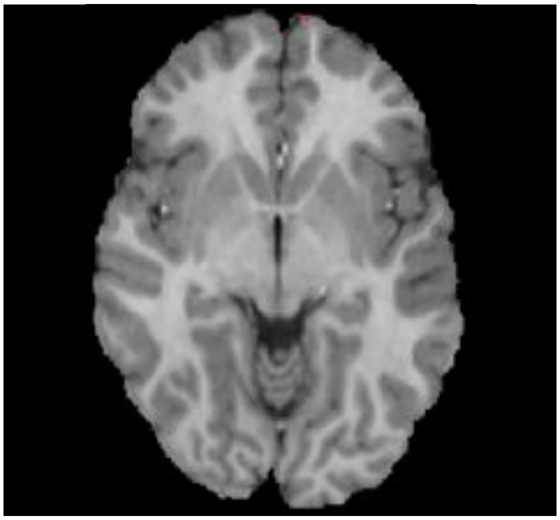

AXIAL

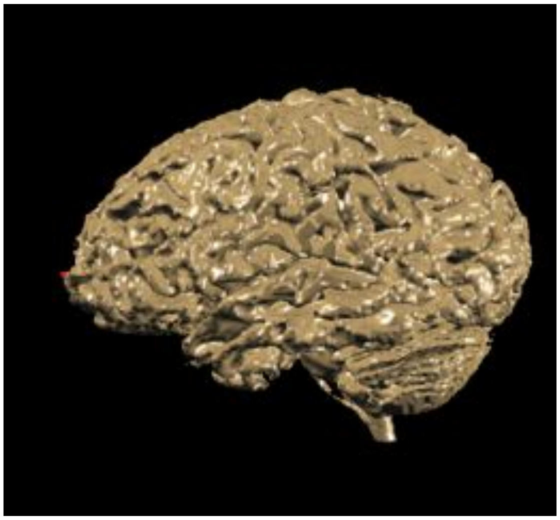

3D

Occipital Pole (OP)

Definition: The most posterior point of the occipital lobe of the cerebrum.

Location: Use the coronal view to identify the most posterior slice of the occipital lobe. (*Note: Look for the posterior-most slice where neural tissue is visible. Be cognizant of residual dura.*) If there is more than one pixel of visible neural tissue, place the landmark in the center of the neural tissue mass. Confirm landmark placement using the axial and sagittal slices.

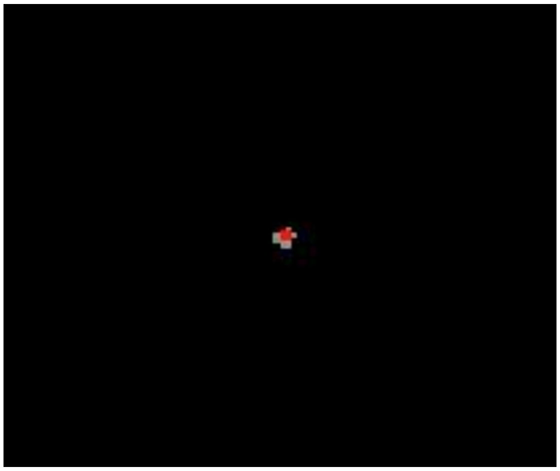

CORONAL

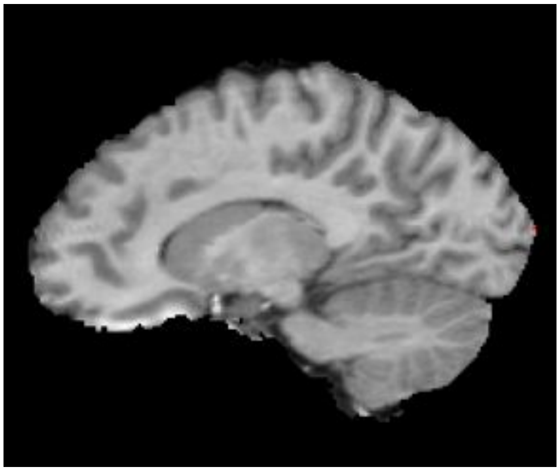

SAGITTAL

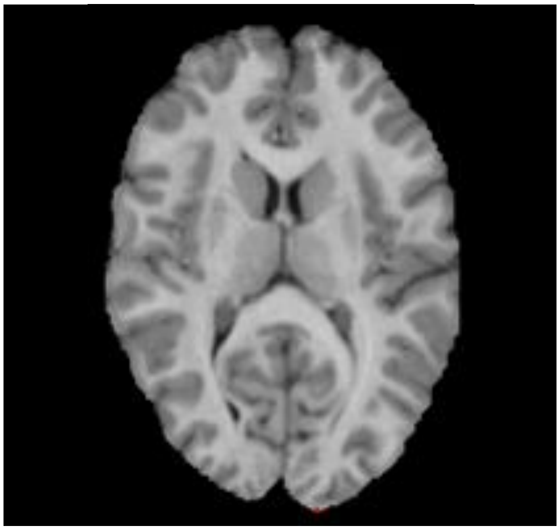

AXIAL

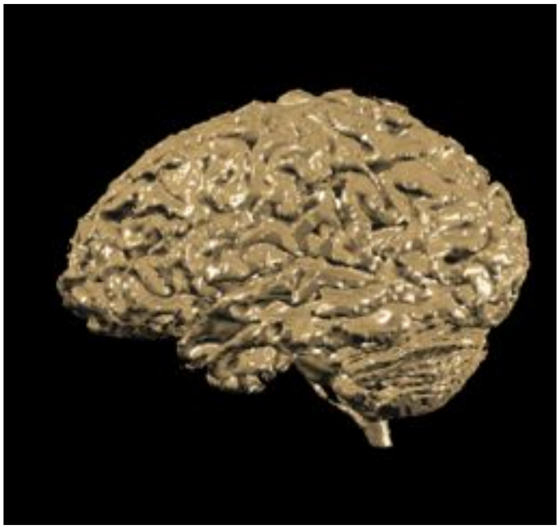

3D

Temporal Pole (TP)

Definition: The most anterior point of the temporal lobe of the cerebrum.

Location: Use the coronal view to identify the most anterior slice of the temporal lobe. (*Note: Look for the anterior-most slice where neural tissue is visible. Be cognizant of residual dura.*) If there is more than one pixel of visible neural tissue, place the landmark in the center of the neural tissue mass. Confirm landmark placement using the axial and sagittal slices.

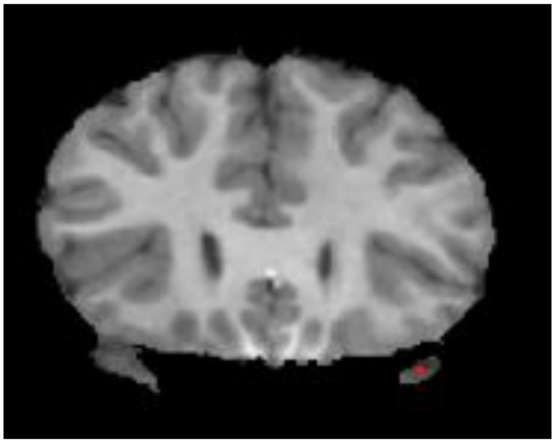

CORONAL

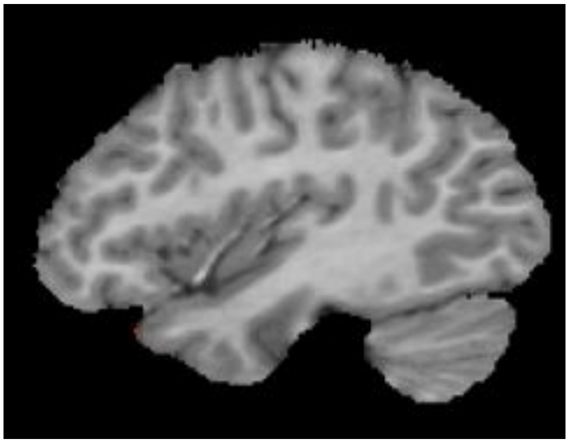

SAGITTAL

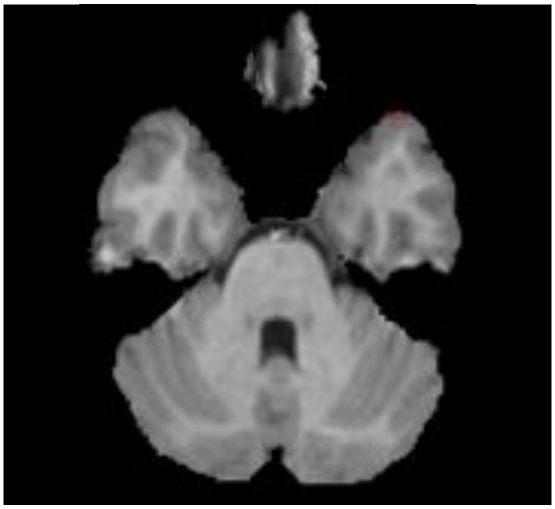

AXIAL

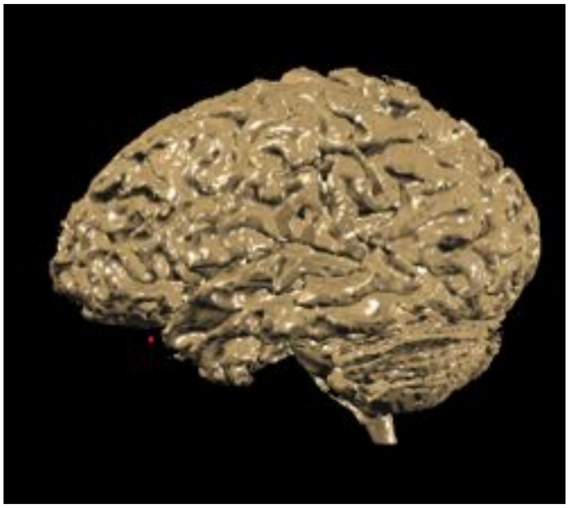

3D

Central Sulcus / Lateral Sulcus (CL)

Definition: The projected intersection of the central sulcus with the lateral sulcus.

Location: 1. Identify the central sulcus using the 3D projection. Place a temporary landmark at the inferior termination of the central sulcus.

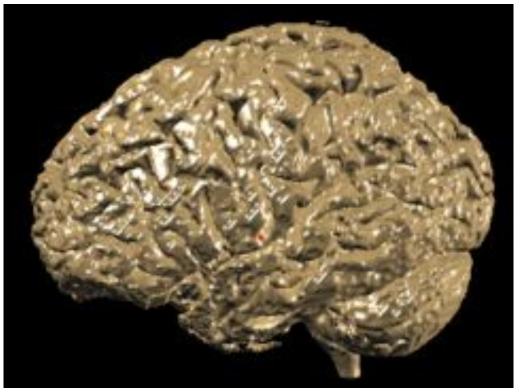

2. Switch to the sagittal view. Scroll laterally to the most lateral slice where the termination of the central sulcus is visible. Neural tissue surrounding the central sulcus (it usually forms a cap around the sulcus) should still be visible. Place the landmark at the termination of the sulcus in this view.

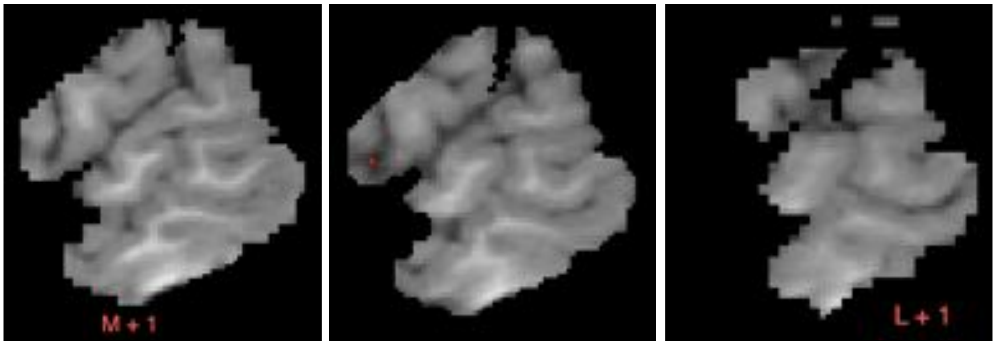

3. Switch to the coronal view. Move the landmark to the lateral sulcus on this coronal slice. The landmark should be placed on the surface (i.e., you may need to move the landmark medial or lateral as well as inferior).

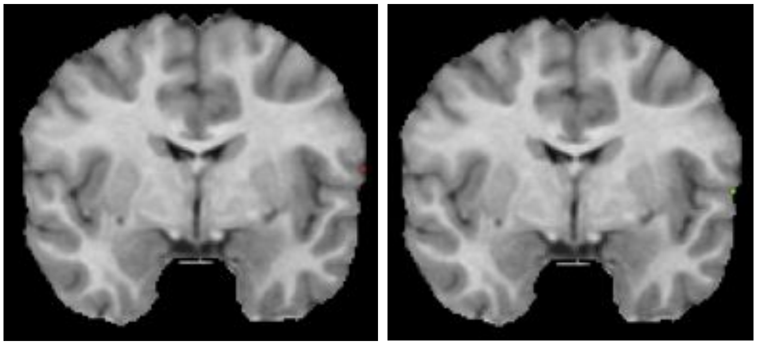

4. Double-check on the 3D view that the landmark is located at the projected intersection of the central s. and lateral s.

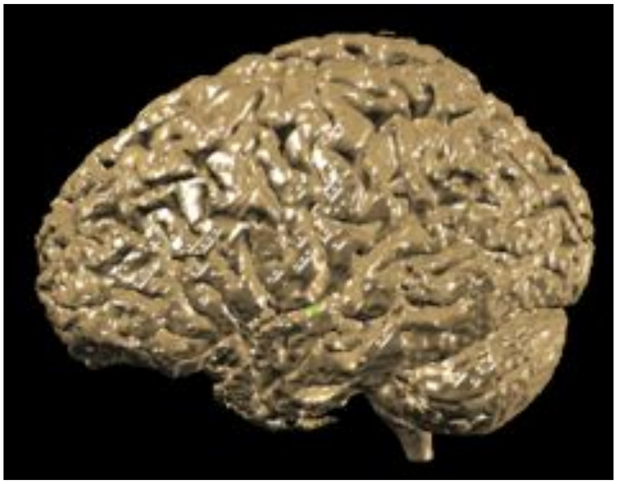

Central Sulcus – Most Superior Point (CS)

Definition: The most superior point of the central sulcus.

Location: 1. Identify the central sulcus using the 3D projection. Place a temporary landmark at the superior termination of the central sulcus.

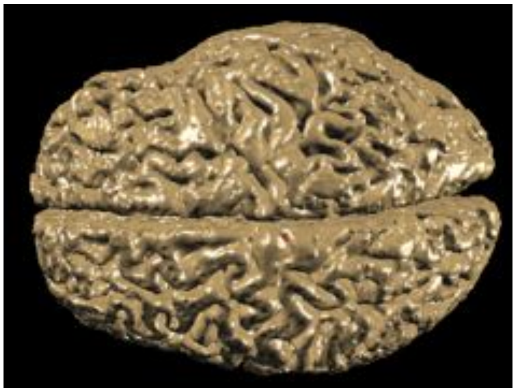

2. Switch to the axial view. Scroll through the slices until you get to the most superior slice where the central sulcus is still visible. Neural tissue should still be visible on either side of (or even forming a cap around) the sulcus. Place a landmark at the most medial point of the sulcus.

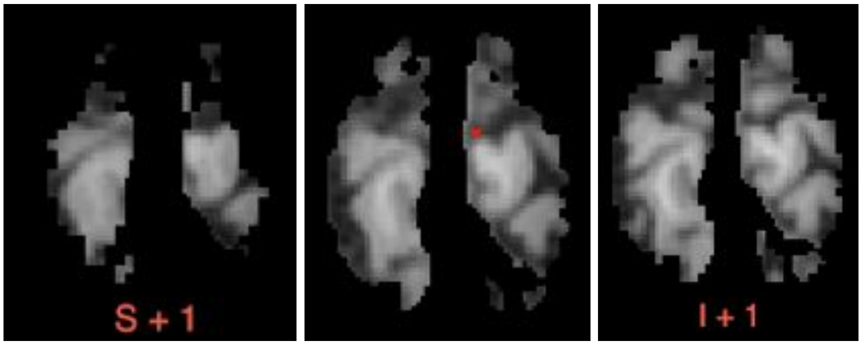

If the sulcus forks at its superior terminus, choose the branch that is visible on the most superior axial slice. If both branches of the sulcus are visible to an equal degree on the most superior axial slice, choose the anterior sulcus.

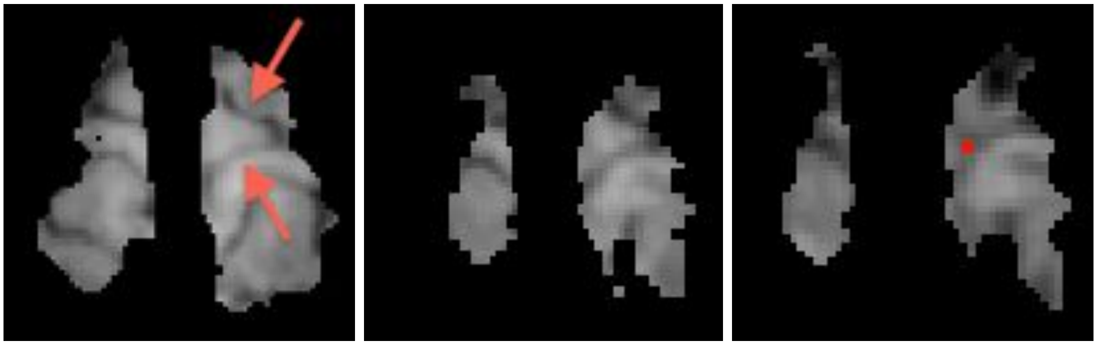

3. Double-check on the 3D view that the landmark is located at the superior-most point of the central sulcus.

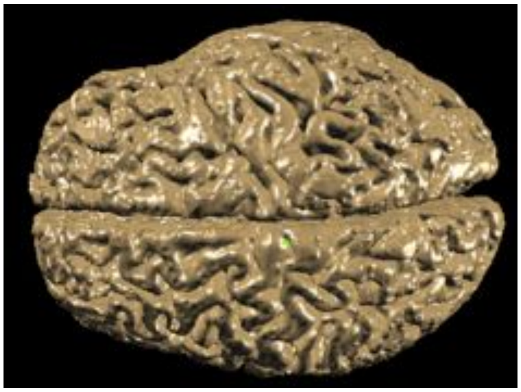

Precentral Sulcus / Superior Frontal Sulcus (PRSF)

Definition: The intersection of the precentral sulcus and the superior frontal sulcus.

Location:

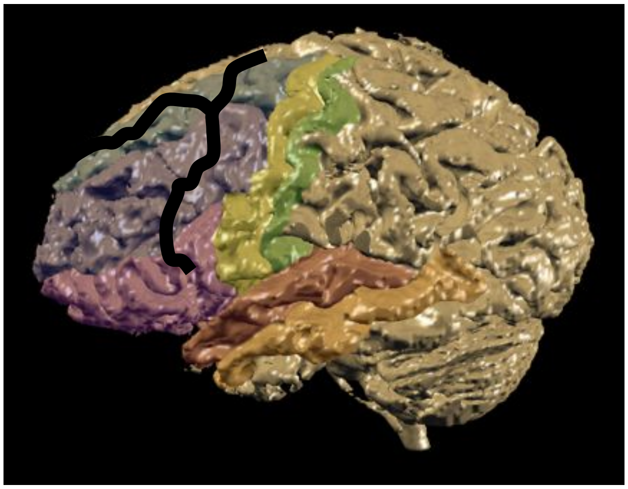

Use the 3D projection to place a landmark at the intersection of the pre-central sulcus with the superior frontal sulcus. Using the axial view, confirm that the landmark has been placed on the most superior slice where this intersection is visible. If you move one slice superiorly, the intersection between these sulci will no longer be visible. Align the landmark with the anterior margin of the pre-central sulcus where it intersects with the superior frontal sulcus.

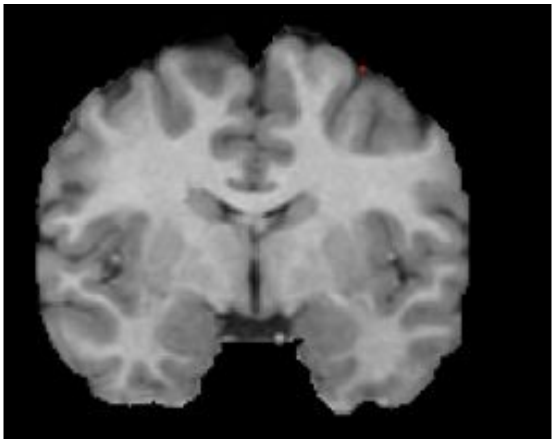

CORONAL

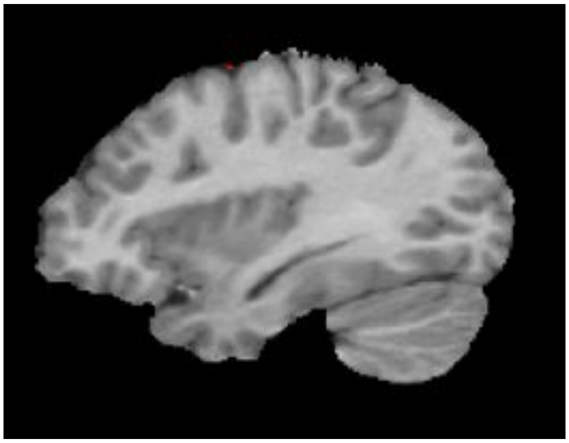

SAGITTAL

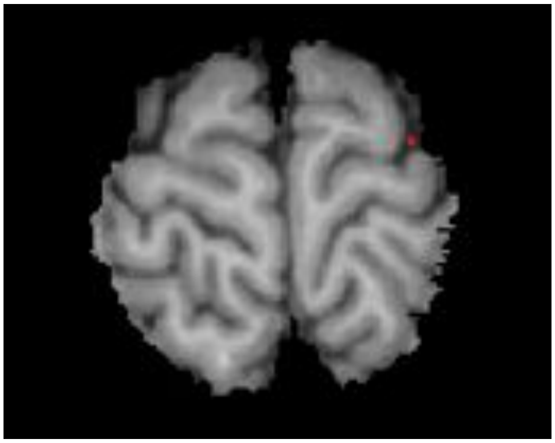

AXIAL

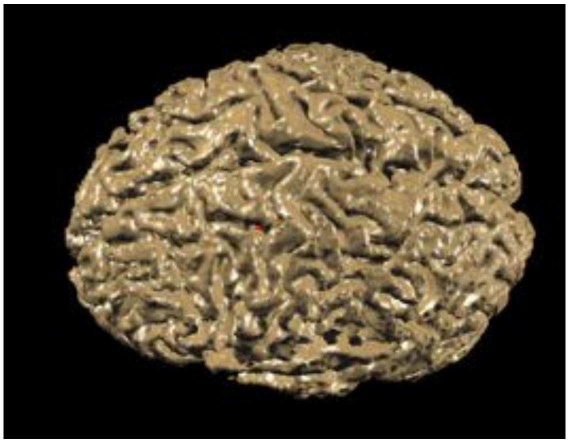

3D

Precentral Sulcus / Inferior Frontal Sulcus (PRIF)

Definition: The intersection of the precentral sulcus with the inferior frontal sulcus.

Location:

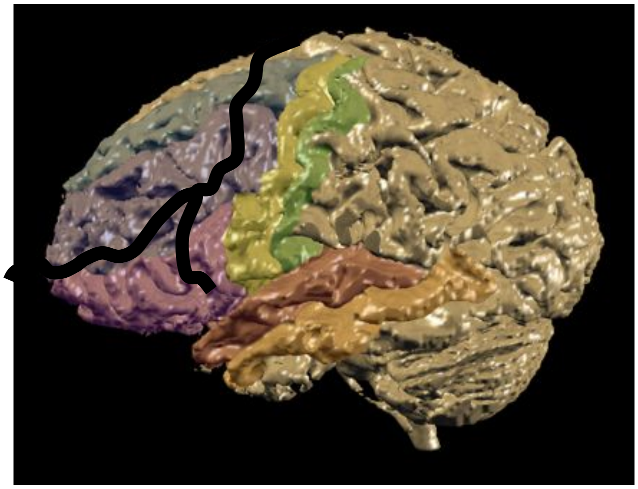

(1) Using the 3D projection, identify the precentral sulcus (see "tips for identifying the precentral sulcus" for help) and then trace it from its superior to inferior termination.

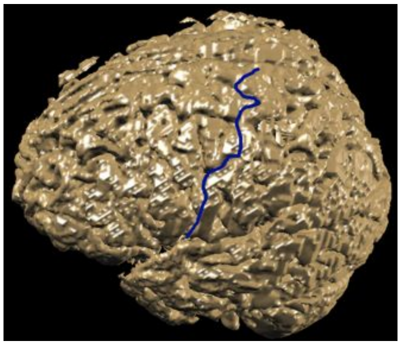

2) Using the 3D projection, identify the inferior frontal sulcus and then trace it from anterior to posterior. Stop at the *first point* when the inferior frontal sulcus intersects with the precentral sulcus. Place the landmark here.

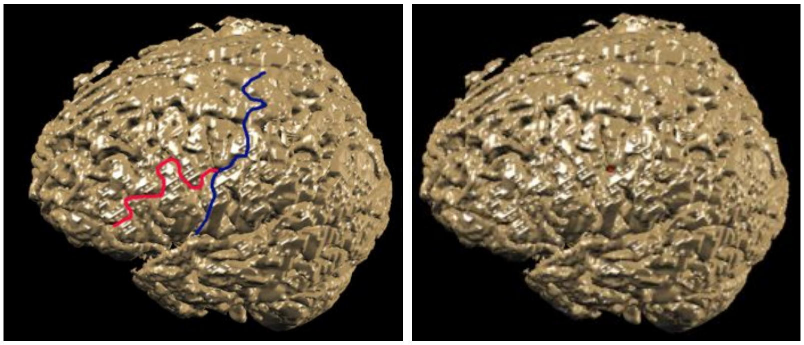

(3) Using either the axial or coronal view, move the landmark out to the surface.

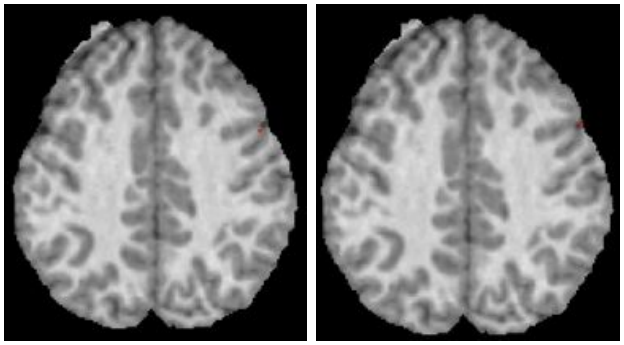

(4) Switch to the sagittal view and adjust your landmark using both the sagittal view and the 3D projection as a guide.

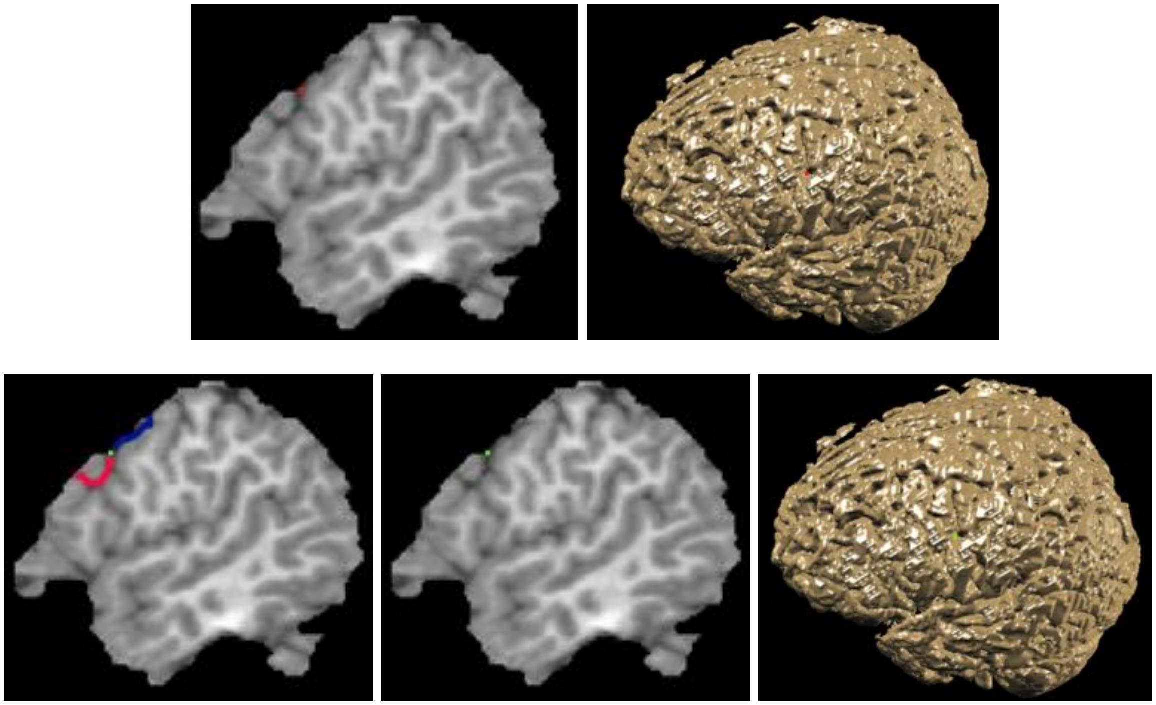

Opercular Sulcus (OPE)

<u>Definition</u>: The superior termination of the opercular sulcus. The opercular sulcus delineates the boundary between the pars opercularis and the pars triangularis in the inferior frontal gyrus.

<u>Location</u>:

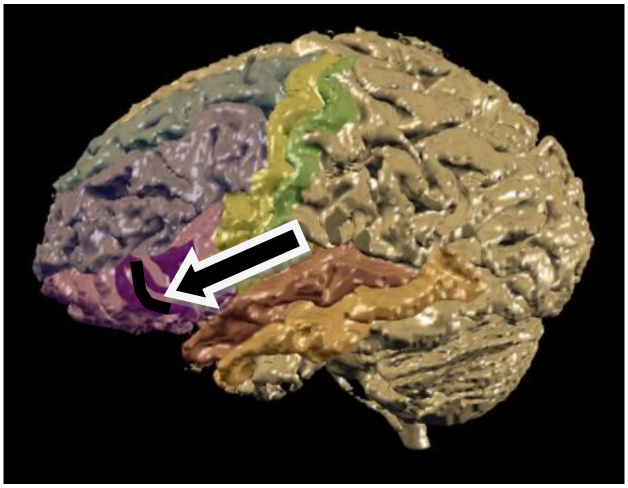

(1) Make sure to identify the precentral sulcus first. The opercular sulcus is the sulcus anterior to the precentral sulcus. The opercular s. is <u>derived from</u> the lateral sulcus.

(2) Use the 3D view to identify the precentral s. Next, identify the opercular s. Place a temporary landmark on the opercular sulcus. Sometimes the sulcus may have a questionable

branch where you're not sure if it ends more inferiorly or more superiorly. If you extract the surface at a high threshold, this can help you to determine whether the branch is part of the opercular s. or derived from the inferior frontal gyrus. If the branch is derived from the inferior frontal sulcus, it is NOT the opercular sulcus. The inferior frontal gyrus should form a cap around the termination of the opercular s.

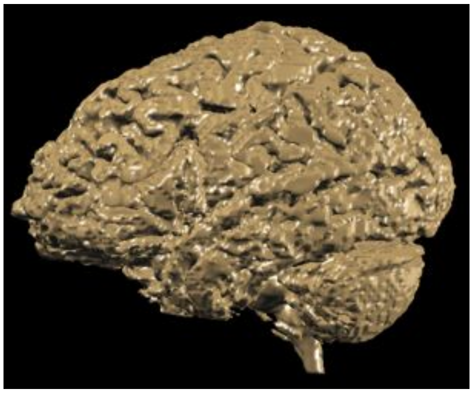

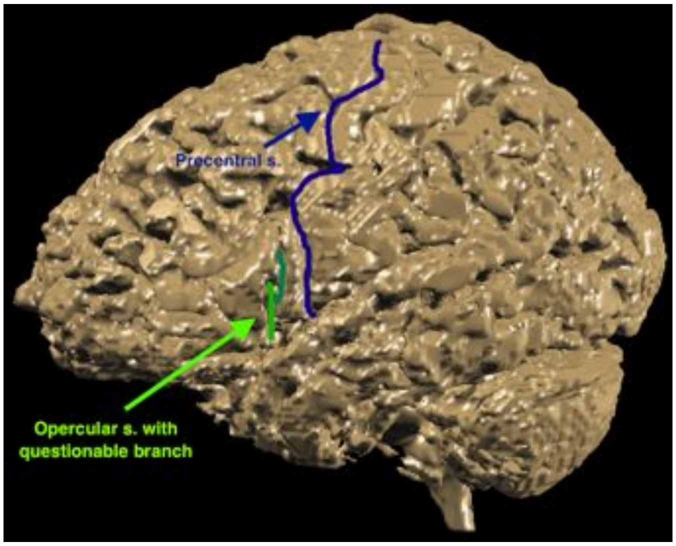

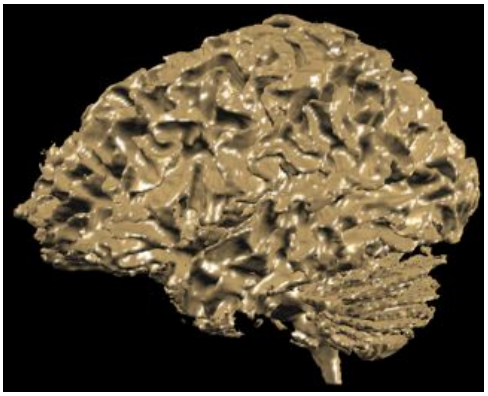

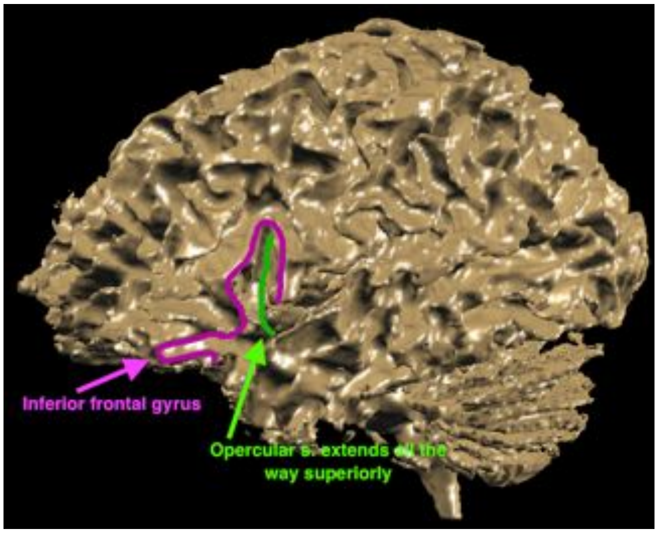

(3) Switch to the sagittal view. Scroll through the sagittal slices to make sure that you are on the slice where the superior-most point of the opercular s. is visible (if you are too lateral, you may place the landmark in the middle of the sulcus rather than at its superior termination.)

Reposition the landmark so that it is at the end of the opercular sulcus, nestled in the cap formed by the inferior frontal g.

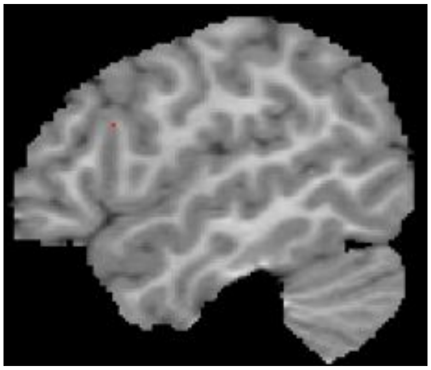

(4) Switch to the axial view or the coronal view to confirm that the landmark is on the surface. If it isn't, follow the sulcus to the surface and then place the landmark to this point.

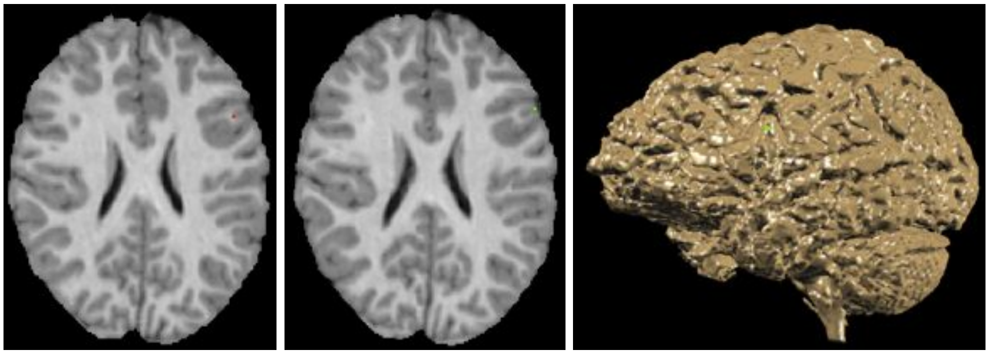

Triangular Sulcus (TRI)

Definition: The superior termination of the triangular sulcus. The triangular sulcus delineates the boundary between the pars triangularis and the pars orbitalis in the inferior frontal gyrus.

Location:

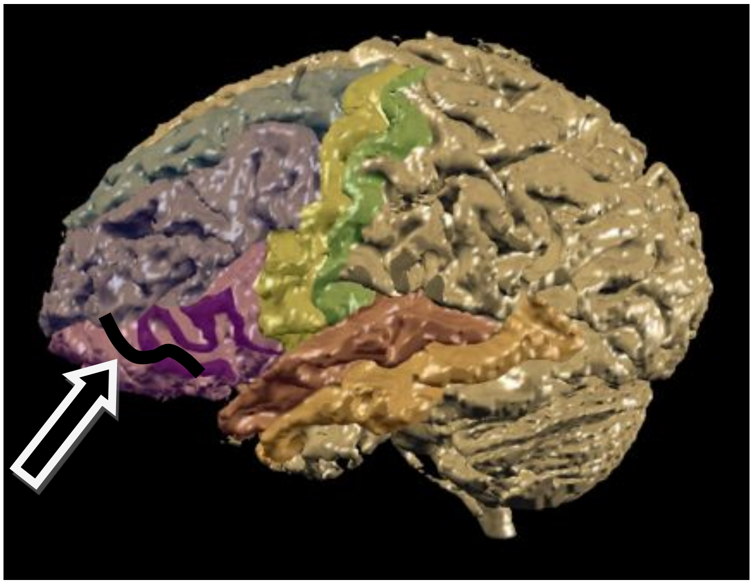

(1) Make sure to identify the precentral s. and opercular s. and to trace the inferior frontal gyrus from anterior to posterior before proceeding. The triangular s is derived from the lateral sulcus. It is the sulcus anterior to the opercular s and forms the other part of the M. Be careful – often the inferior frontal gyrus loops in such a way as there is a sulcus that may appear to derive from the lateral sulcus and is directly anterior to the opercular sulcus but is NOT the triangular s. When you look at the 3D projection, there is no tissue underlying the triangular s – it is derived directly from the lateral s. The inferior frontal gyrus should form a cap around the superior termination of the triangular s.

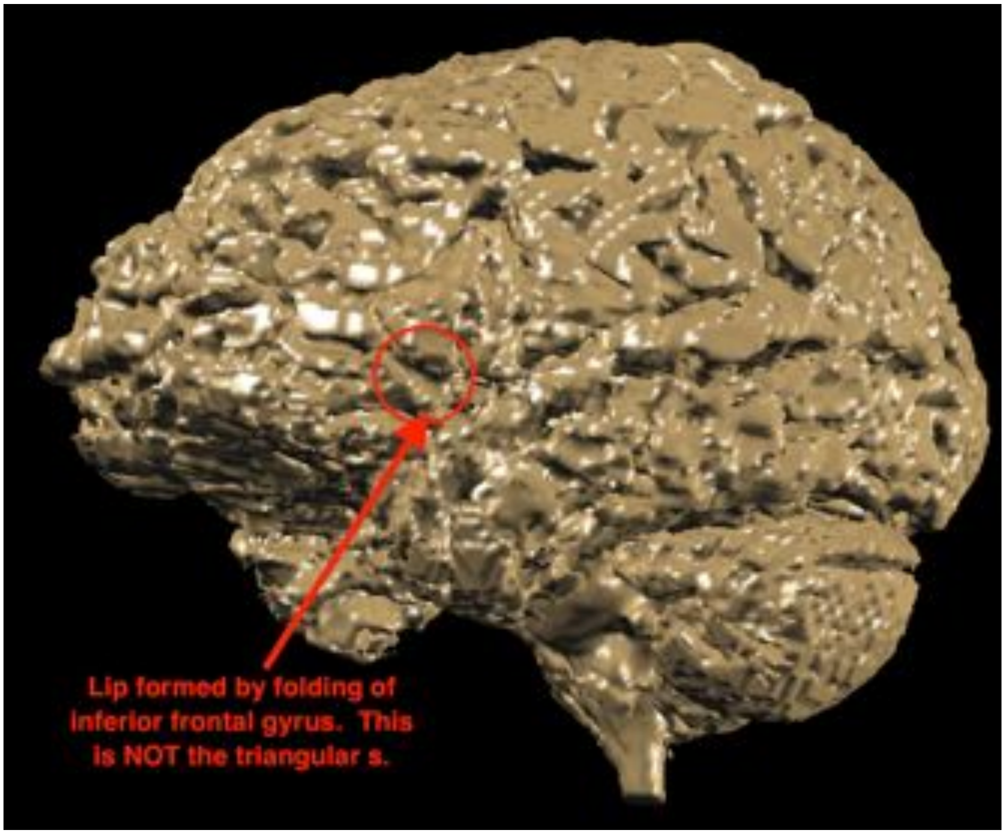
Lip formed by folding of
inferior frontal gyrus. This
is NOT the triangular s.

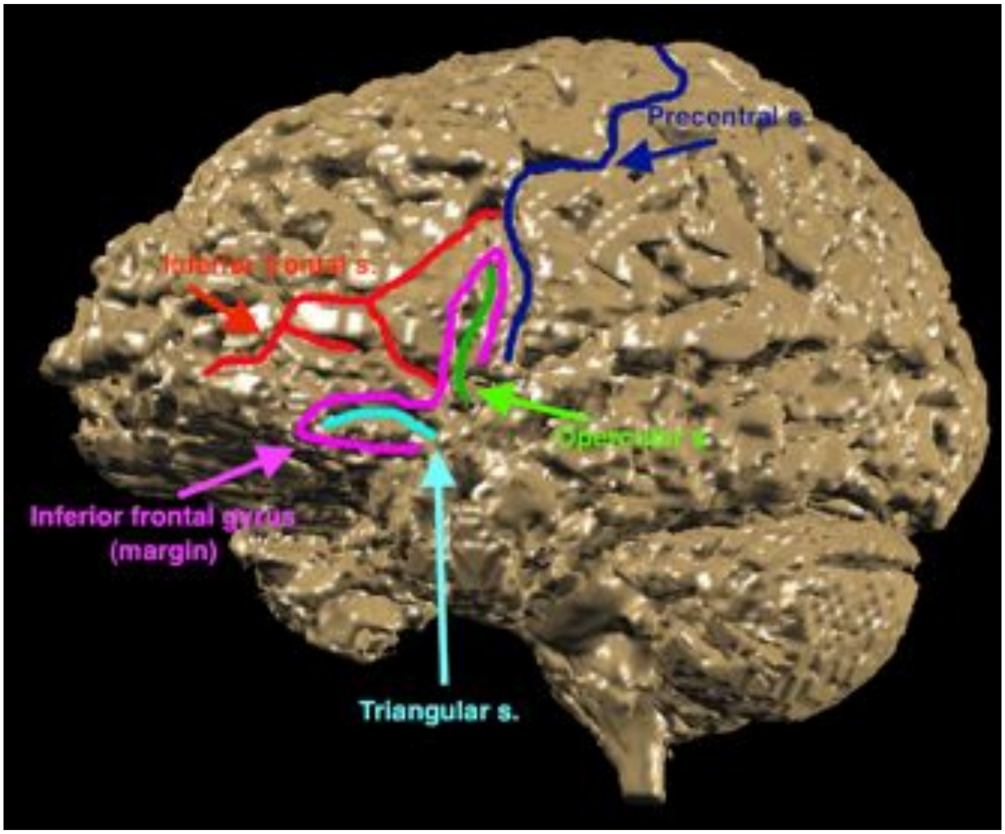
Precentral s.
Inferior frontal gyrus
(margin)
Triangular s.

(2) Using the 3D projection, place a temporary landmark at the superior termination of the triangular s. Then switch to the sagittal view. Scroll through the sagittal slices to make sure that you are on the slice where the anterior-most point of the triangular s. is visible (if you are too lateral, you may place the landmark in the middle of the sulcus rather than at its anterior termination.) Reposition the landmark so that it is at the end of the triangular s., nestled in the cap formed by the inferior frontal gyrus.

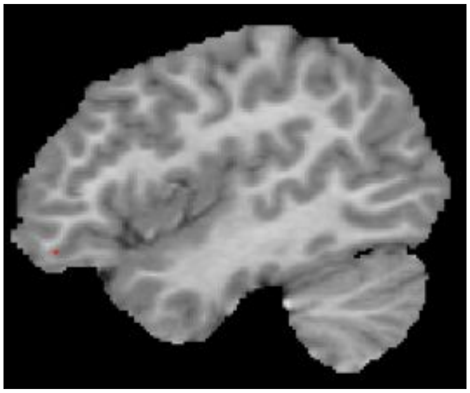

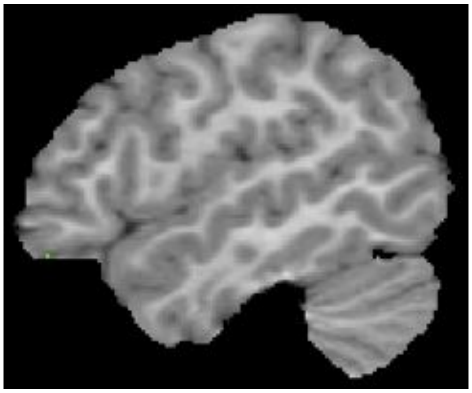

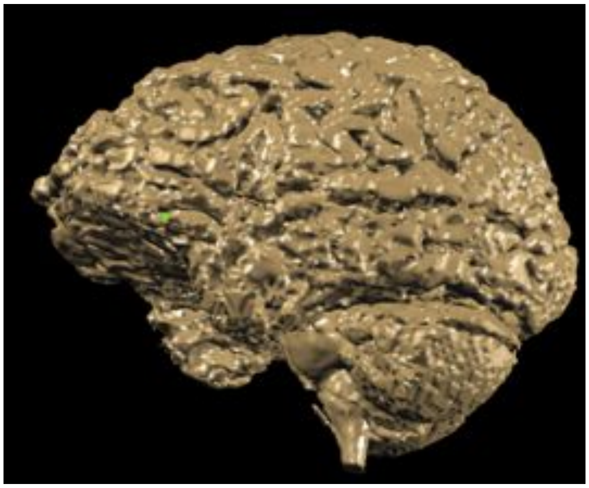

(3) Switch to the axial view or the coronal view to confirm that the landmark is on the surface. If it isn't, follow the sulcus to the surface and then place the landmark to this point.

Posterior Termination of the Superior Temporal Sulcus (STP)

<u>Definition</u>: The most posterior point of the superior temporal sulcus before it splits into the superior temporal ascending branch and the superior temporal posterior branch.

<u>Location</u>:

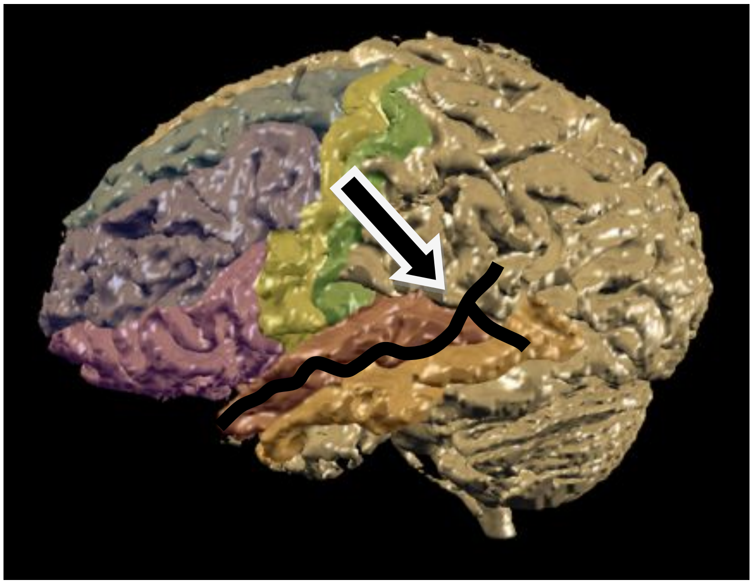

(1) Identify the lateral sulcus by tracing it from anterior to its posterior termination.

(2) Identify the superior temporal sulcus and trace it from anterior to posterior. If there is a fork, take the superior branch.

(3) Identify the point when the STP turns superiorly. This will be posterior to two vertical (parallel) gyri – the anterior of which ends the lateral sulcus. The STP will usually fork here so one branch will turn superiorly to run posterior to the 2nd gyrus and the other branch will continue running posteriorly.

Example 1:

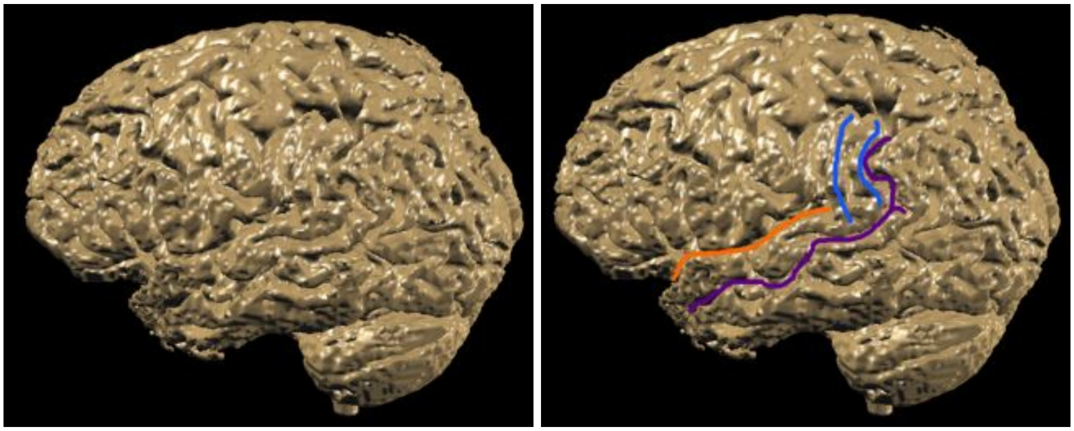

Example 2:

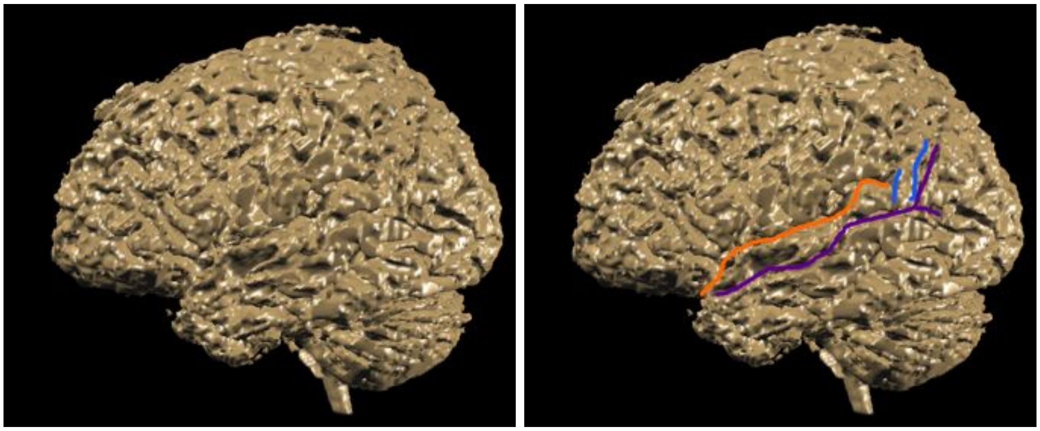

Example 3:

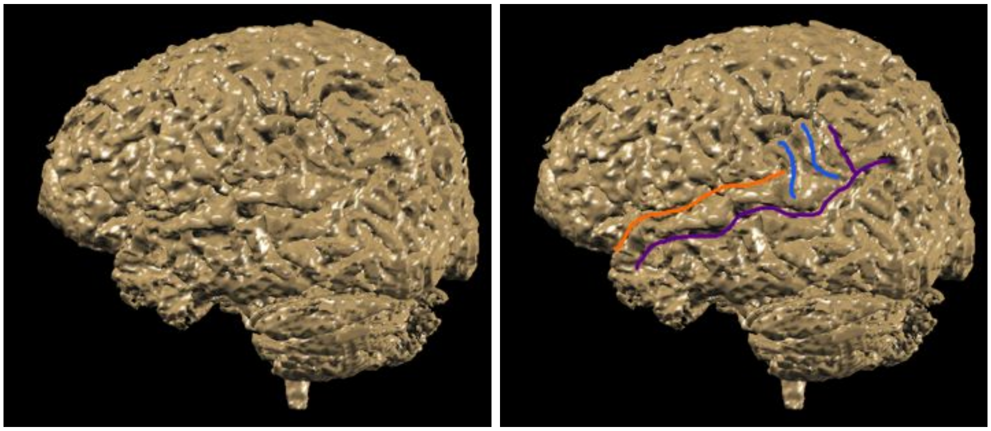

(4) Using the 3D projection, place the landmark at the point where the STP branches and/or turns superiorly. This point will be postero-inferior to the 2nd vertical gyrus.

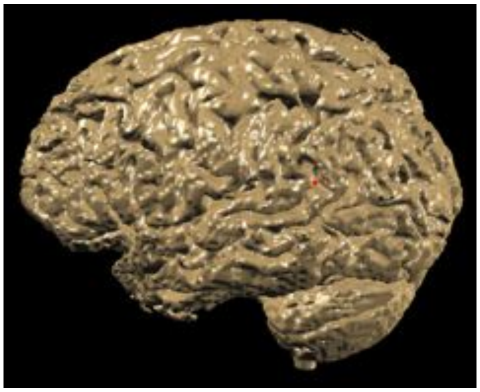

(5) Switch to the sagittal view. Reposition the landmark so that it is at the point where the STP branches and/or turns superiorly (you may have to scroll laterally/medially to visualize this sulcus better).

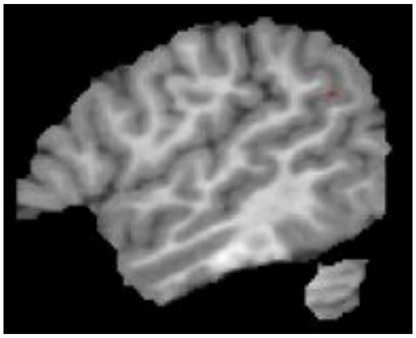

(6) Switch to the axial or coronal view. Trace the sulcus to the surface and place the landmark on the surface.

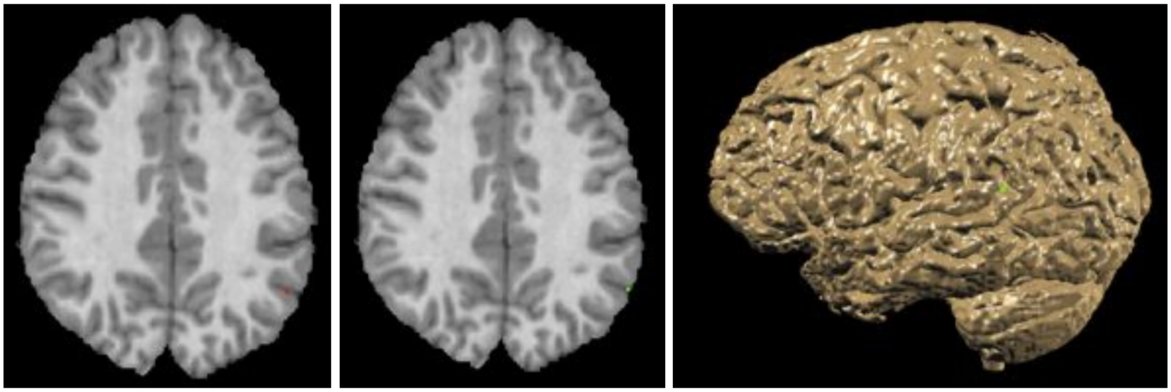

Parietooccipital Sulcus – Most Superior Point (POS)

Definition: The superior termination of the parietooccipital sulcus on the (locally-defined) mid-sagittal slice of the cerebrum.

Location: Identify the mid-sagittal slice of the cerebrum using the coronal and axial views as a guide. Follow the parietooccipital sulcus superiorly along the occipital lobe. Place the landmark at the superior-most point of the parietooccipital sulcus along the occipital lobe. If a researcher would like to landmark the brain bilaterally, the researcher should average the parieto-occipital s. landmarks from the L and R sides.

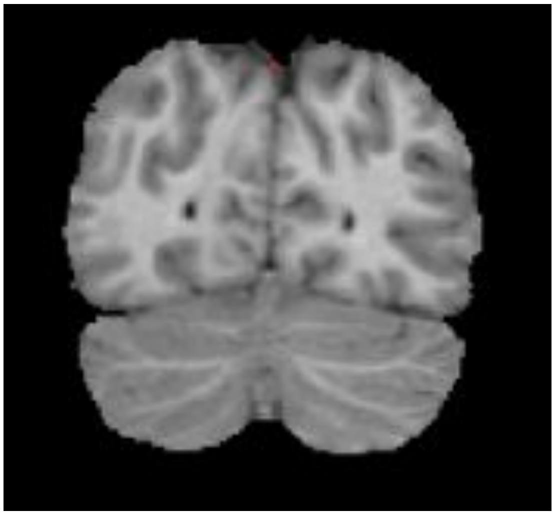

CORONAL

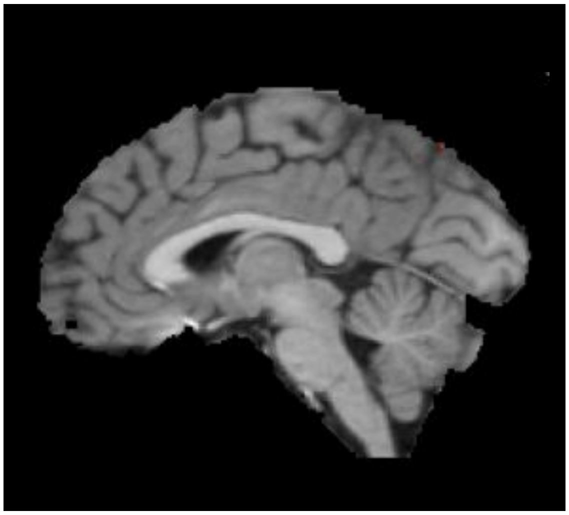

SAGITTAL

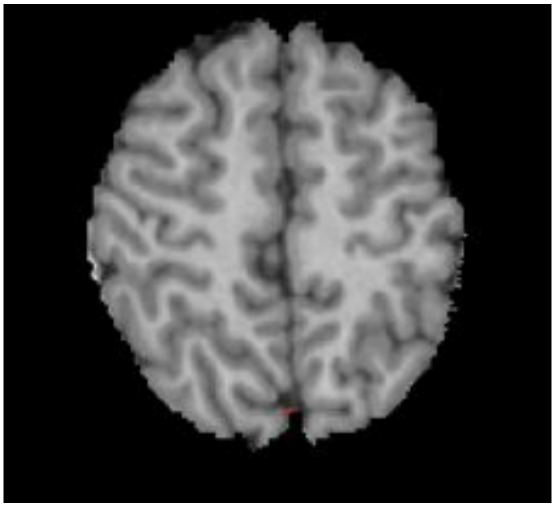

AXIAL

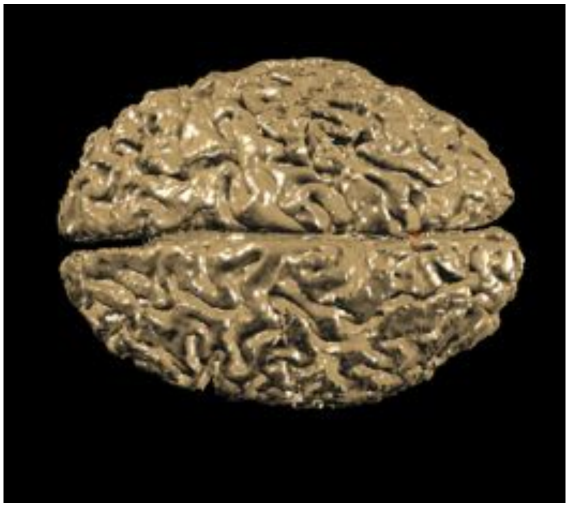

3D

Cerebellum – Lateral Pole (CBL)

Definition: The most lateral point of the cerebellum.

Location: Use the sagittal view to find the most lateral aspect of the cerebellum. If there is more than one pixel of visible cerebellar tissue, place the landmark in the center of the cerebellar tissue mass. Images:

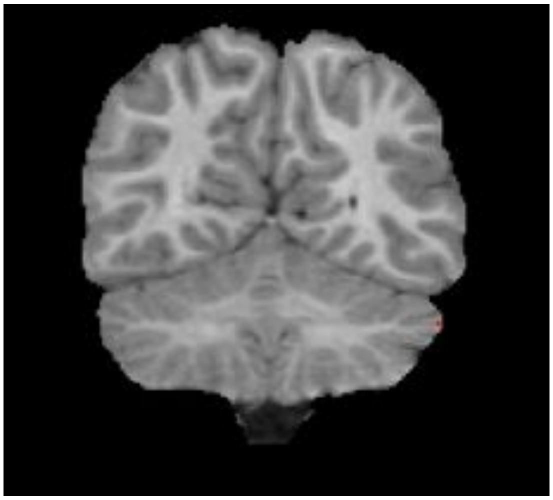

CORONAL

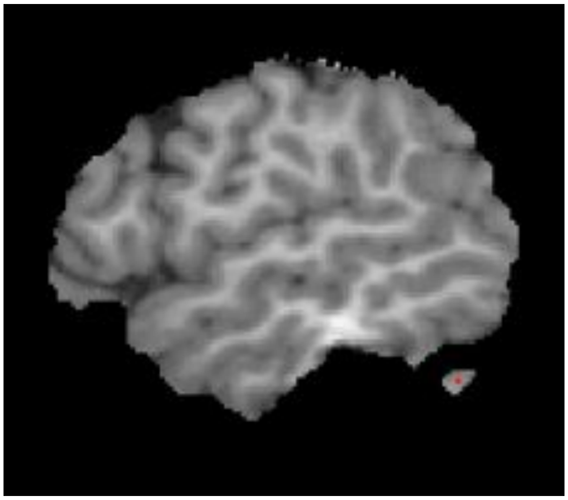

SAGITTAL

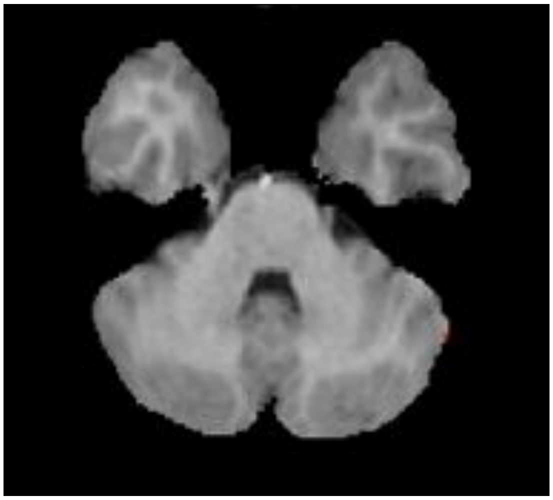

AXIAL

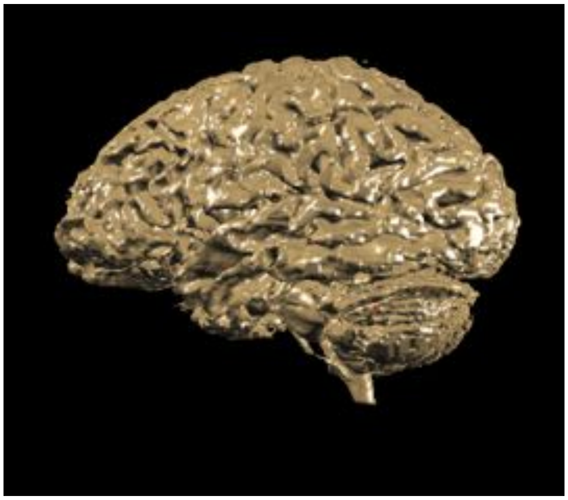

3D

Cerebellum – Midsagittal Inferior (CBI)

Definition: The most inferior aspect of the cerebellum on the (locally-defined) midsagittal slice of the cerebellum.

Location: Identify the midsagittal slice of the cerebellum using the coronal and axial views as a guide. Define the midsagittal slice of the cerebellum locally to the landmark (not the entire cerebellum). Place the landmark on the most inferior point of the vermis. If other cerebellar tissue is visible, disregard it. If there is a row of inferior points, place the landmark in the middle of the row.

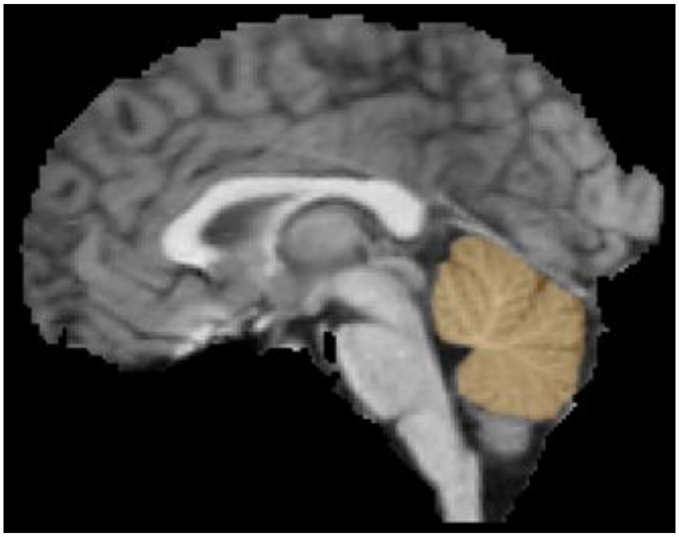

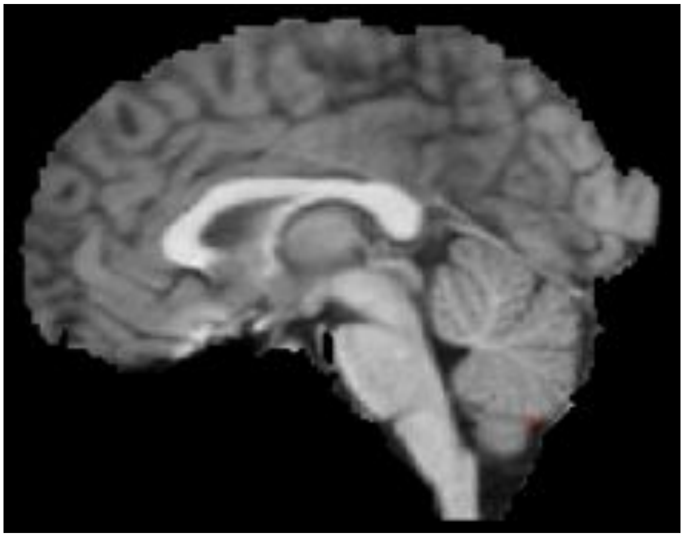

Cerebellum – Midsagittal Posterior (CBP)

Definition: The most posterior aspect of the cerebellum on the (locally-defined) midsagittal slice of the cerebellum.

Location: Identify the midsagittal slice of the cerebellum using the coronal and axial views as a guide. Define the midsagittal slice of the cerebellum locally to the landmark (not the entire cerebellum). Place the landmark on the most posterior point of the vermis. If other cerebellar tissue is visible, disregard it. Remember – if there is a column of posterior points, place the landmark in the middle of the column.

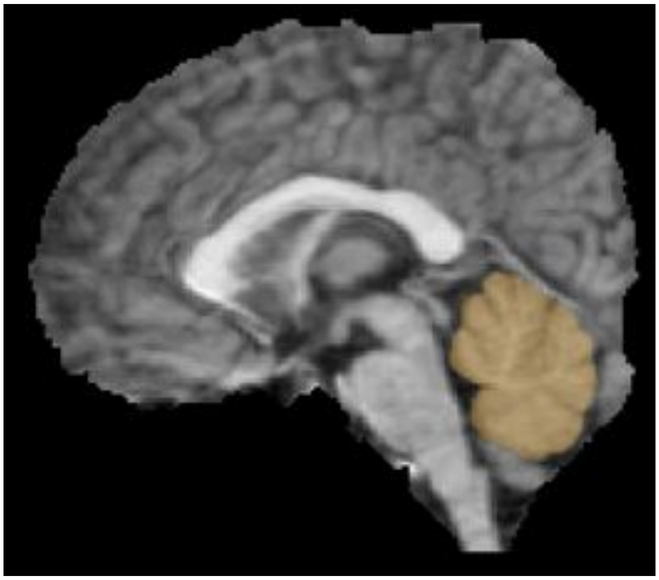

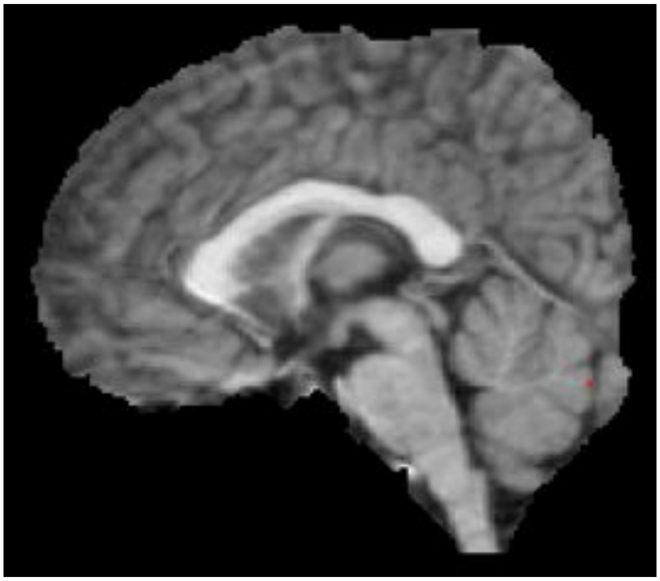

Cerebellum – Midsagittal Superior (CBS)

Definition: The most superior aspect of the cerebellum on the (locally-defined) midsagittal slice of the cerebellum.

Location: Identify the midsagittal slice of the cerebellum using the coronal and axial views as a guide. Define the midsagittal slice of the cerebellum locally to the landmark (not the entire cerebellum). Place the landmark on the most superior point of the cerebellum. If there is more than one pixel, place the landmark in the center of the row.

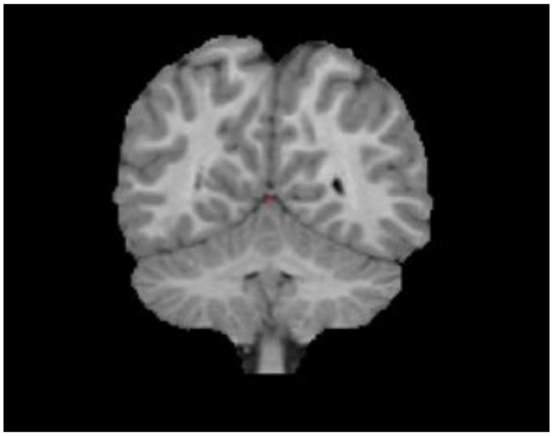

CORONAL

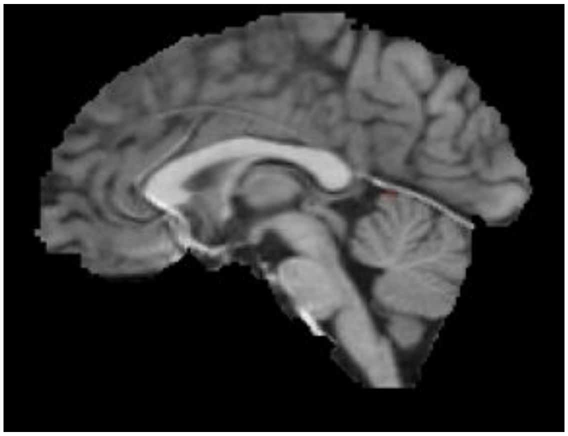

SAGITTAL

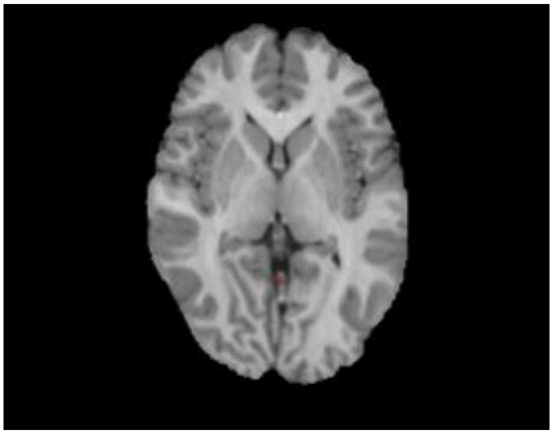

AXIAL

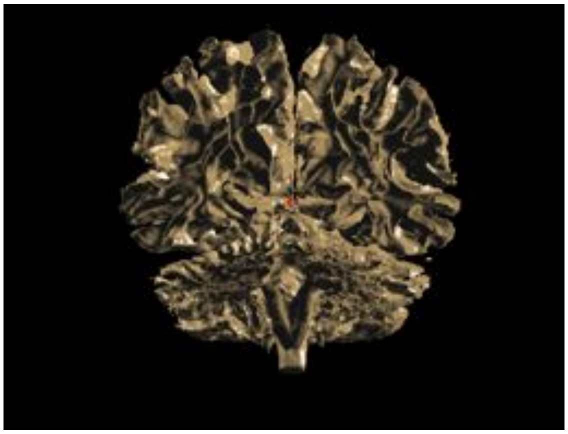

3D

Fourth Ventricle – posterior-most point (FV)

Definition: The most posterior point of the fourth ventricle on the (locally-defined) midsagittal slice of the cerebellum.

Location: Identify the midsagittal slice of the cerebellum using the coronal and axial views as a guide. Define the midsagittal slice of the cerebellum locally to the landmark (not the entire cerebellum). Place the landmark on the most posterior point of the fourth ventricle – at the tip of the triangle.

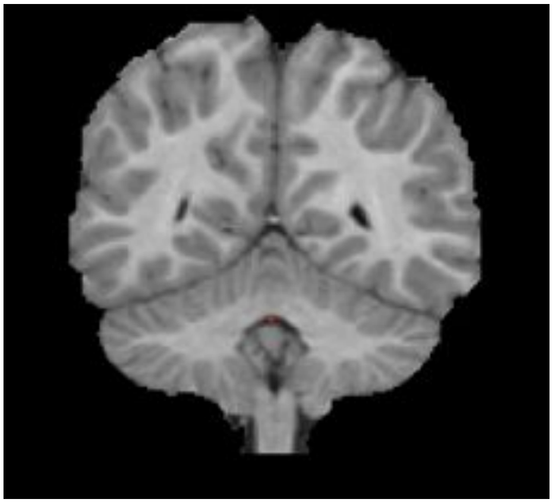

CORONAL

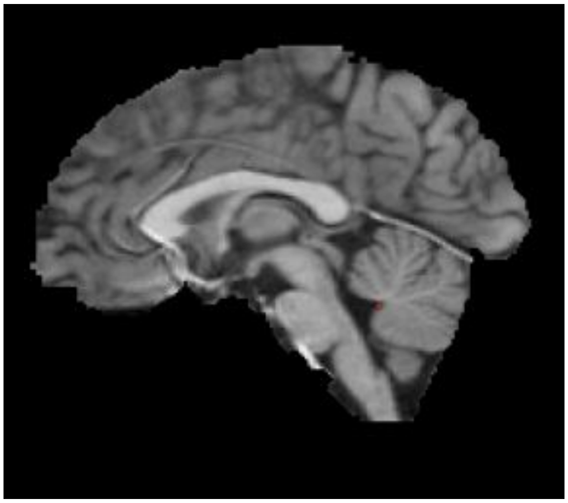

SAGITTAL

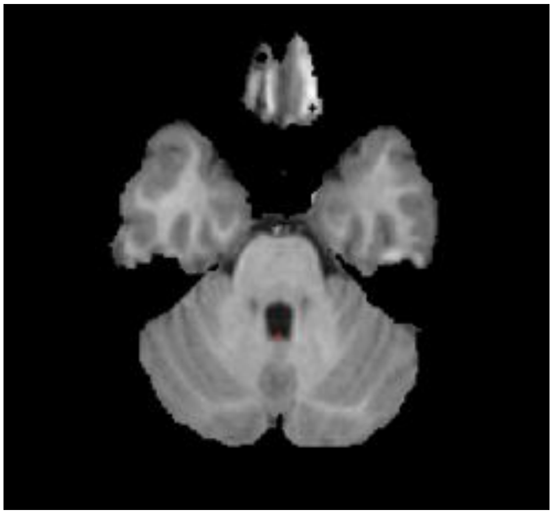

AXIAL

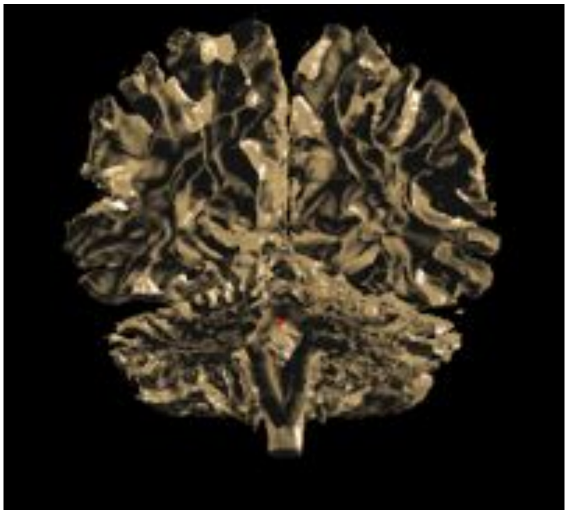

3D

Amygdala (AMYG)

Definition: The centroid of the amygdala.

Location: Use the coronal view to estimate the centroid of the amygdala. Place a temporary landmark. Switch to the axial view and adjust the landmark accordingly. Go back and forth between the coronal and axial slices until the landmark is at the centroid of the amygdala. (Note: If you need help finding a good starting location, scroll through coronal view and find the inferior horn of the lateral ventricle.) Place your first temporary landmark in the center of the amygdala on this slice.)

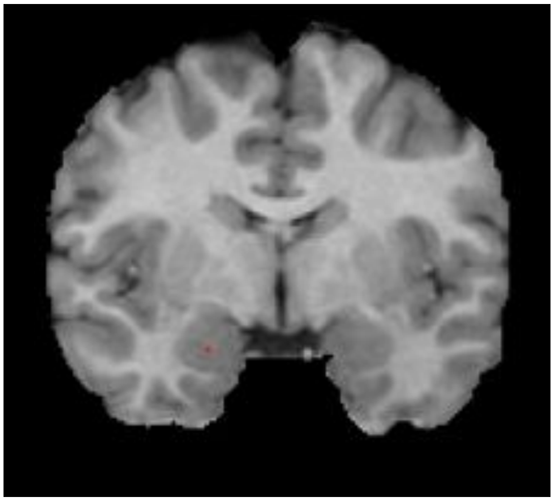

CORONAL

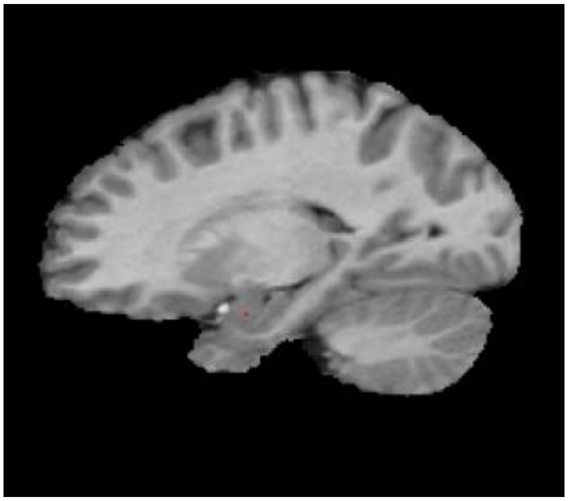

SAGITTAL

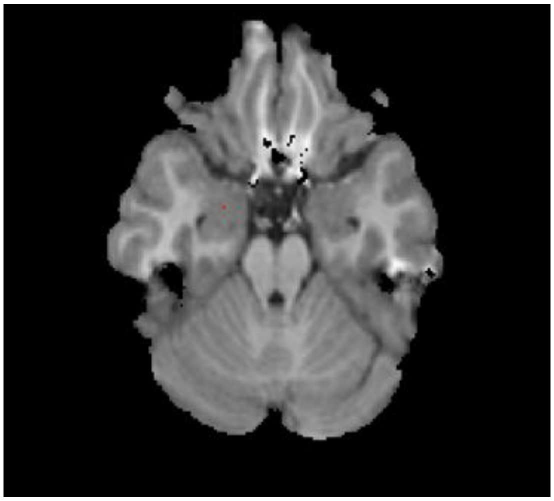

AXIAL

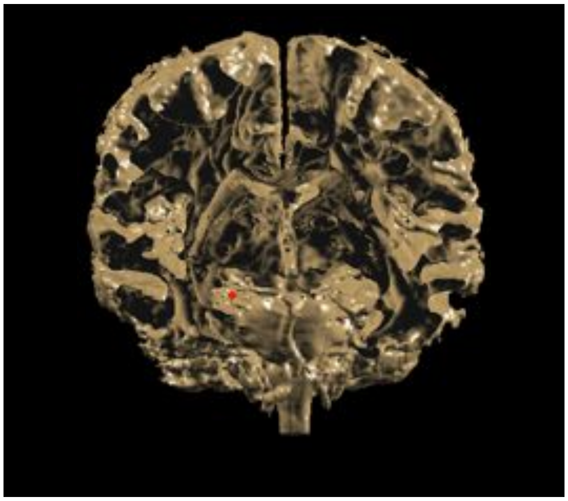

3D

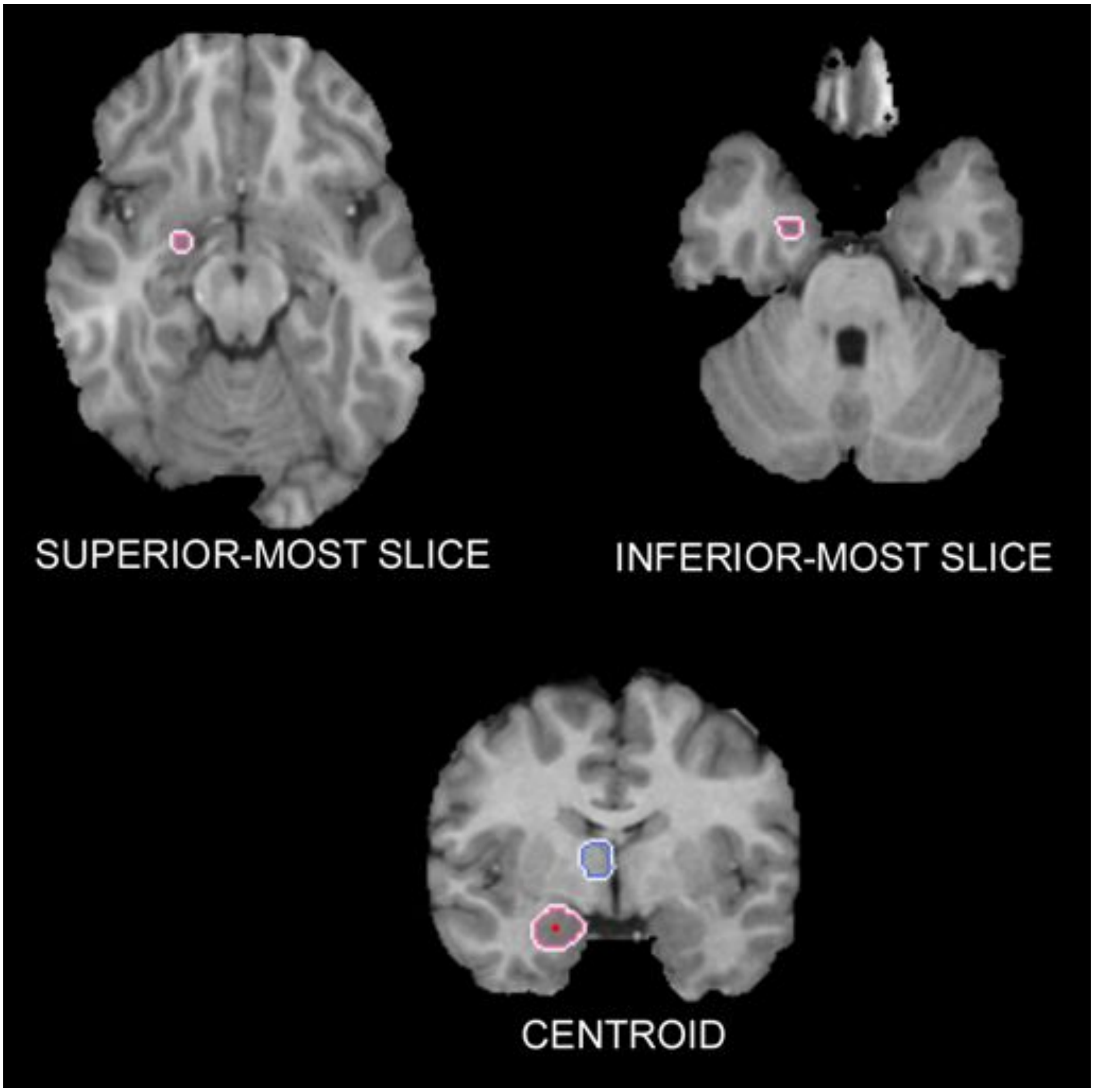
SUPERIOR-MOST SLICE
INFERIOR-MOST SLICE
CENTROID

Caudate Nucleus (CN)

<u>Definition</u>: The centroid of the caudate nucleus.

<u>Location</u>: Use the coronal view to estimate the centroid of the caudate nucleus. Place a temporary landmark. Switch to the axial view and adjust the landmark accordingly. Go back and forth between the coronal and axial slices until the landmark is at the centroid of the caudate nucleus. *(Note: To find a good starting location, scroll through coronal view counting the number slices where the caudate nucleus is visible and divide by two.)*

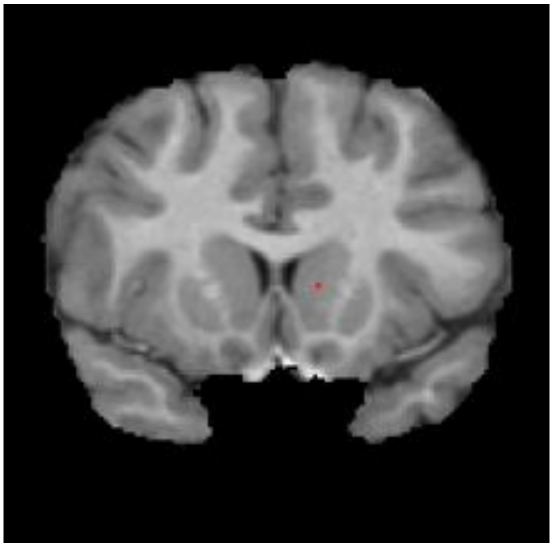

CORONAL

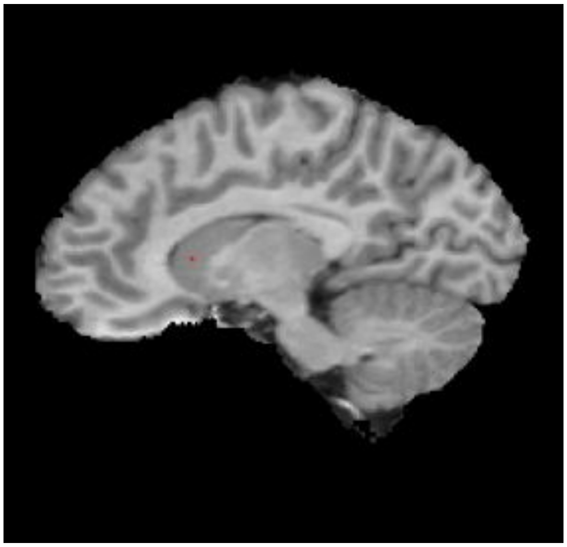

SAGITTAL

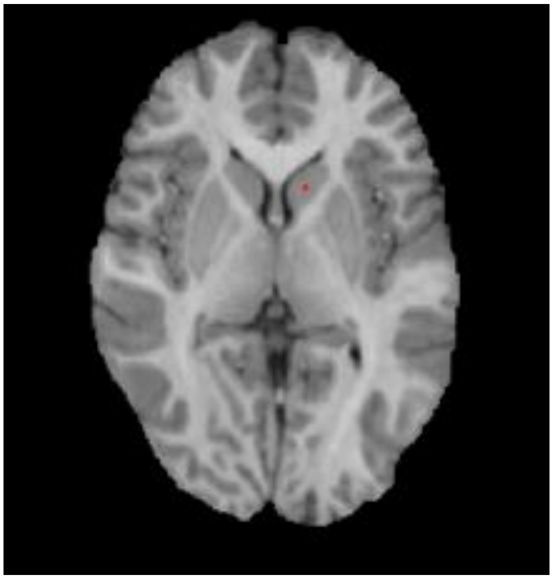

AXIAL

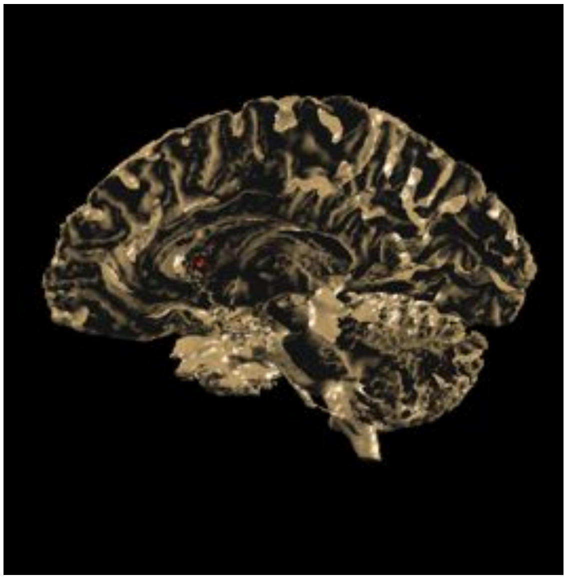

3D

Thalamus – Centroid (THAL)

Definition: The centroid of the thalamus.

Location: Use the coronal view to estimate the centroid of the thalamus. Place a temporary landmark. Switch to the axial view and adjust the landmark accordingly. Go back and forth between the coronal and axial slices until the landmark is at the centroid of the thalamus. *(Note: If you need help finding a good starting location, scroll through coronal view counting the number slices where the thalamus is visible. Divide by two and then go to the halfway point.)*

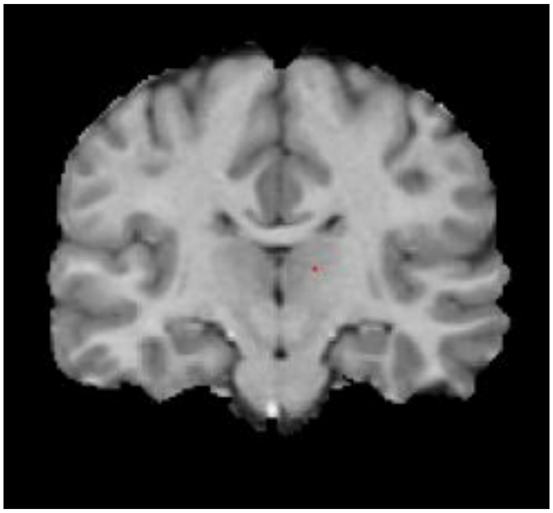

CORONAL

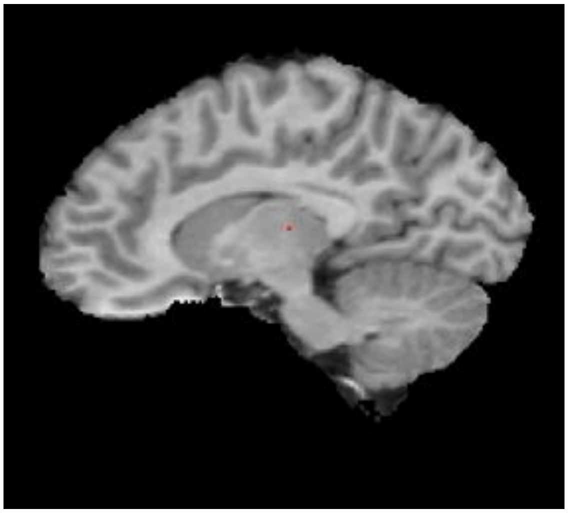

SAGITTAL

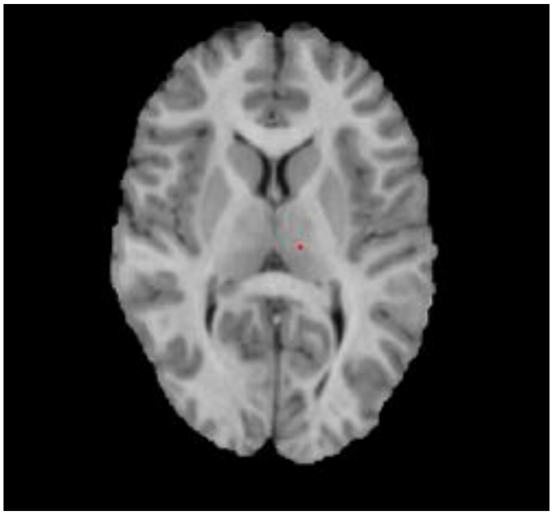

AXIAL

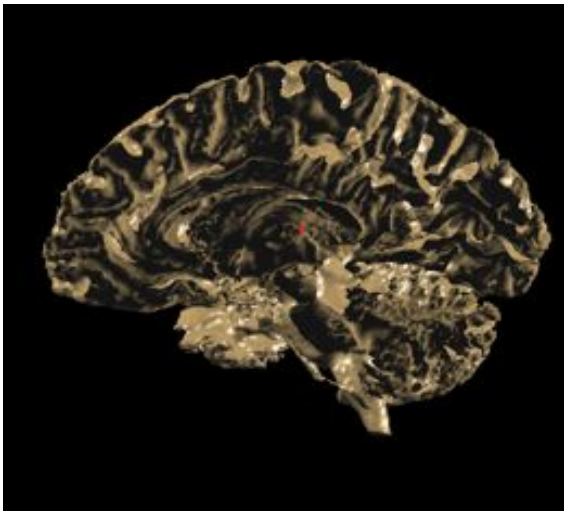

3D

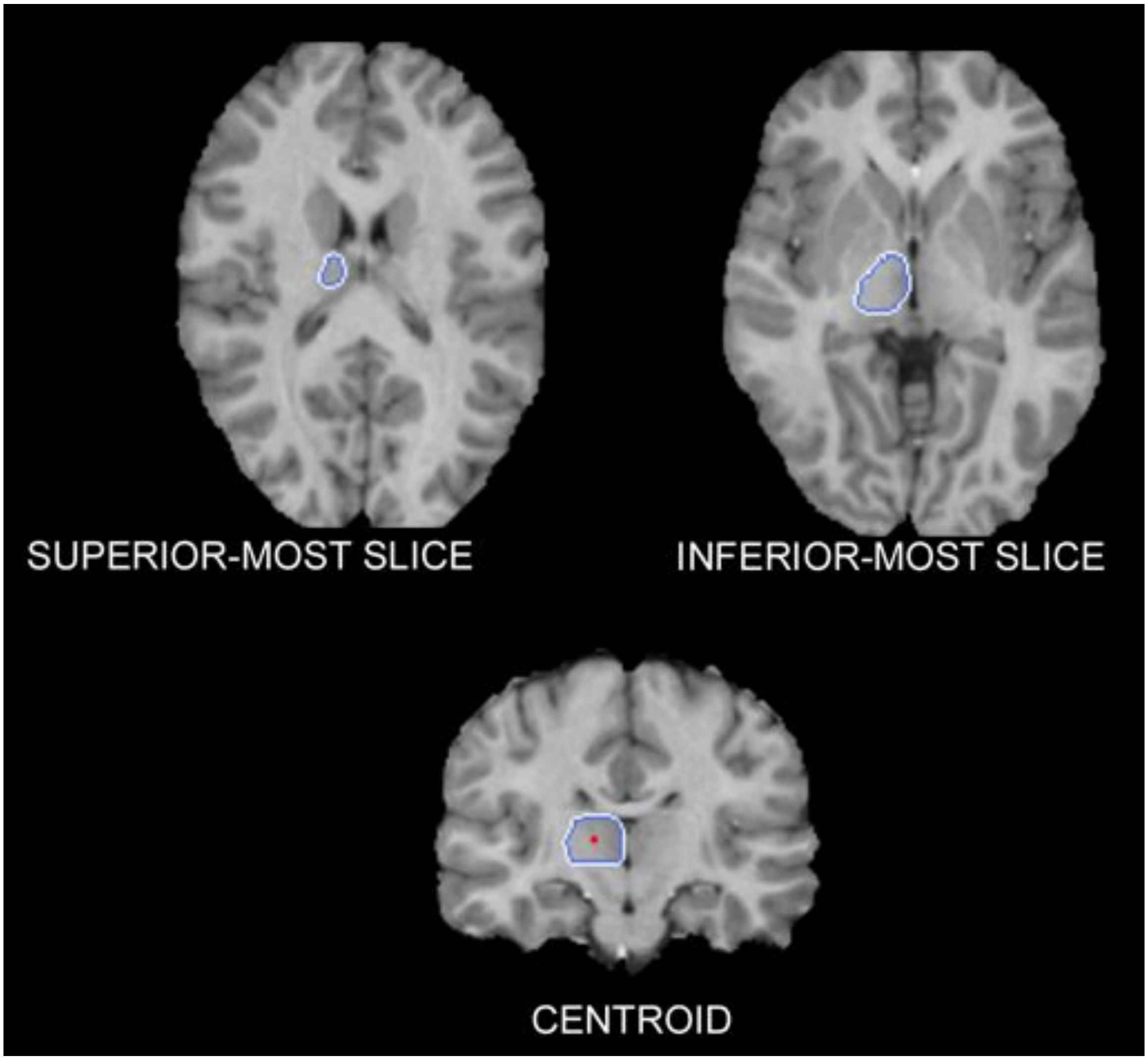
SUPERIOR-MOST SLICE
INFERIOR-MOST SLICE
CENTROID

Corpus Callosum – Genu, Anterior (CCGA)

<u>Definition</u>: The most anterior point of the genu of the corpus callosum.

<u>Location</u>: Define the midsagittal slice of the genu of the CC locally using the coronal and axial views as a guide. The midsagittal slice of the genu of the CC may not be the same slice as the midsagittal slice of the midpoint or splenium of the CC. On the mid-sagittal slice, place the landmark on the most anterior point of the corpus callosum. If there is more than one pixel, place the landmark in the center of the column.

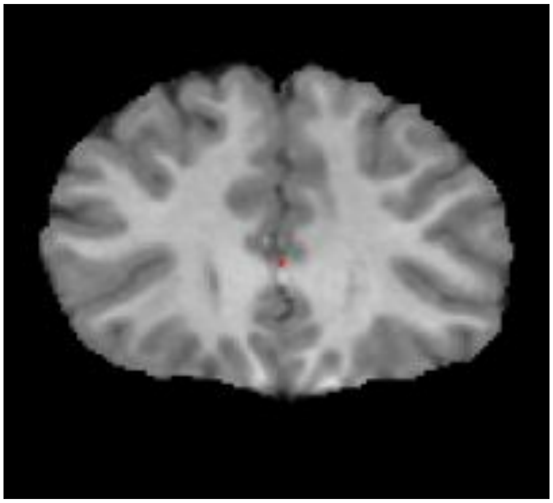

CORONAL

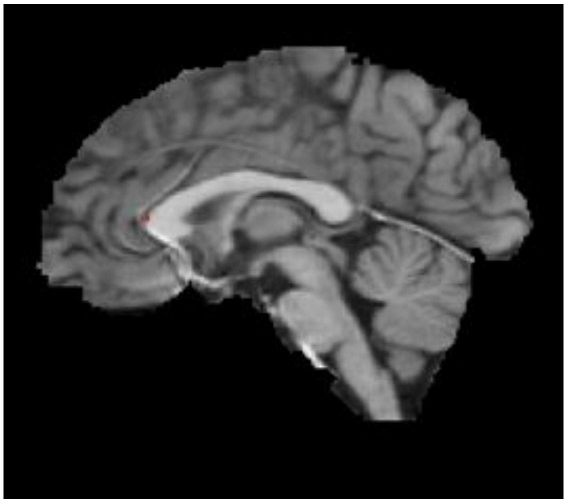

SAGITTAL

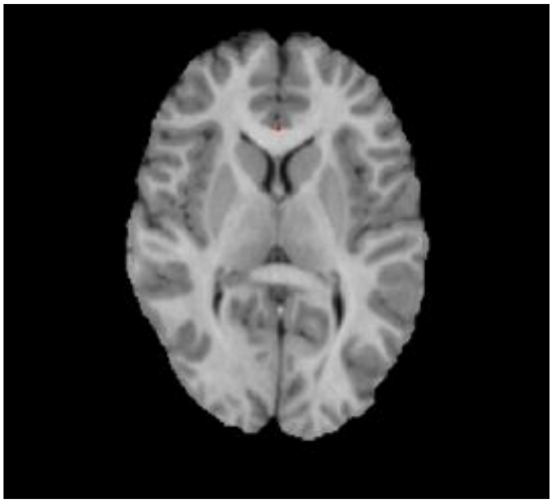

AXIAL

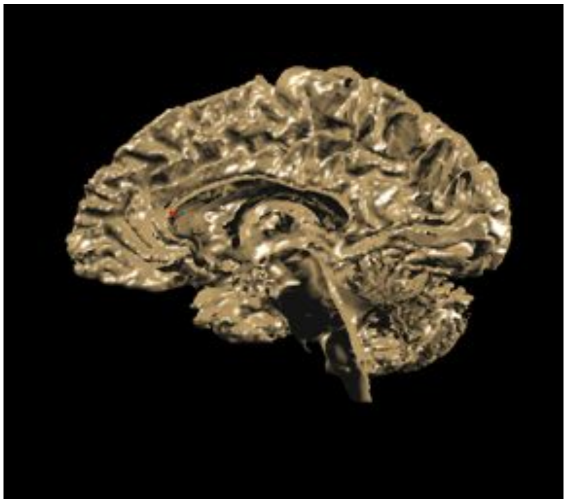

3D

Corpus Callosum – Midpoint, Superior (CCMS)

<u>Definition</u>: The most superior point of the corpus callosum.

<u>Location</u>: Define the midsagittal slice of the superior midpoint of the CC locally using the coronal and axial views as a guide. On the mid-sagittal section, place the landmark on the most superior point of the corpus callosum. If there is more than one pixel in the most superior row, choose the middle pixel.

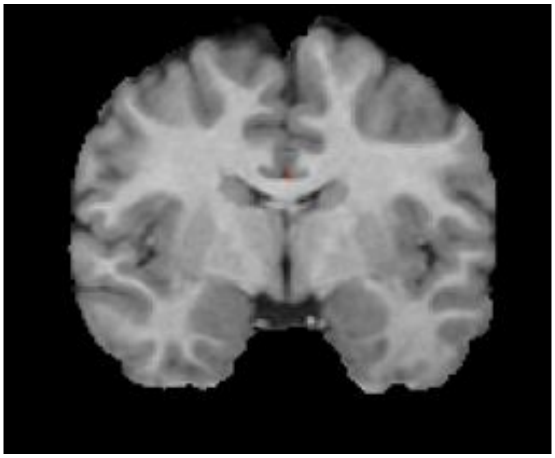

CORONAL

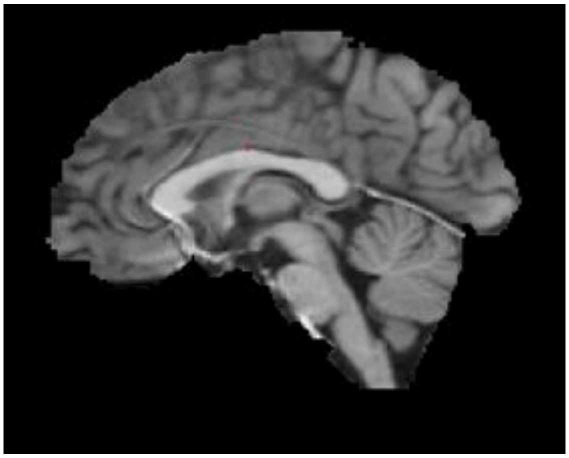

SAGITTAL

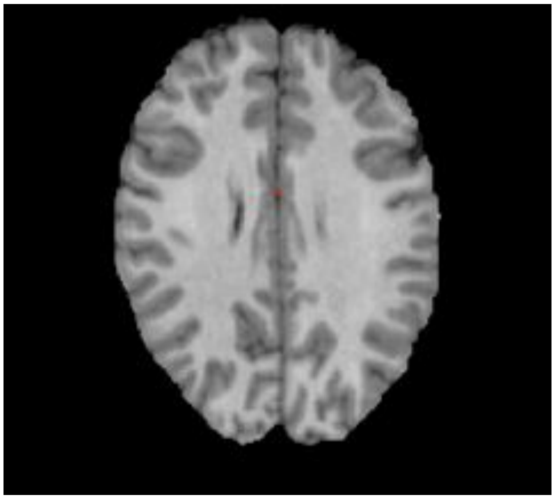

AXIAL

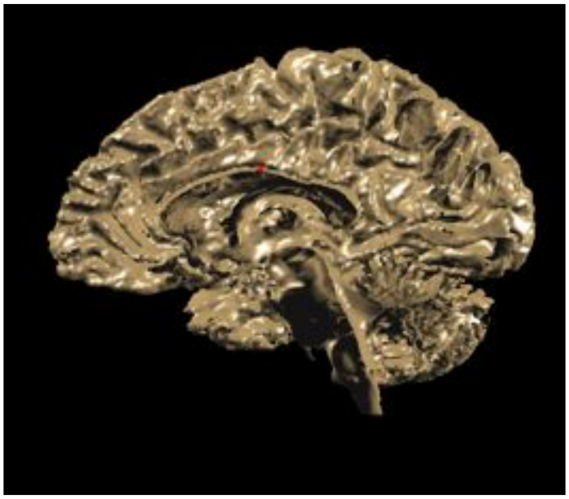

3D

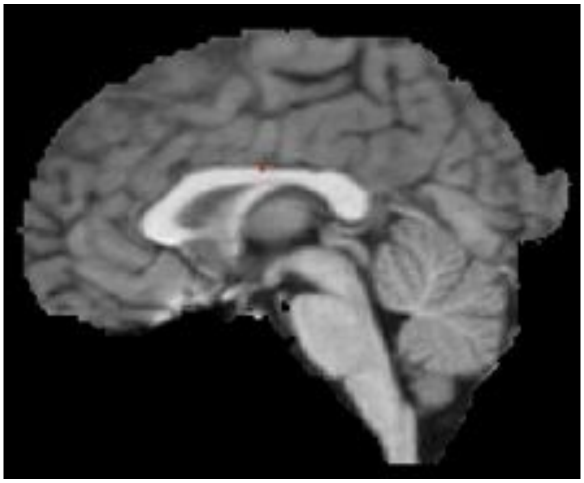

Corpus Callosum – Splenium, posterior (CCSP)

Definition: The most posterior point of the splenium of the corpus callosum.

Location: Define the mid-sagittal plane locally using the coronal and axial slices. Using the mid-sagittal section, place the landmark on the most posterior point. If there is more than one pixel, place the landmark in the middle of the column.

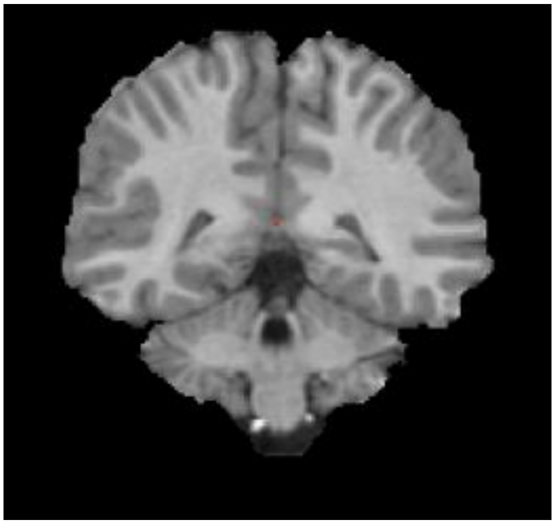

CORONAL

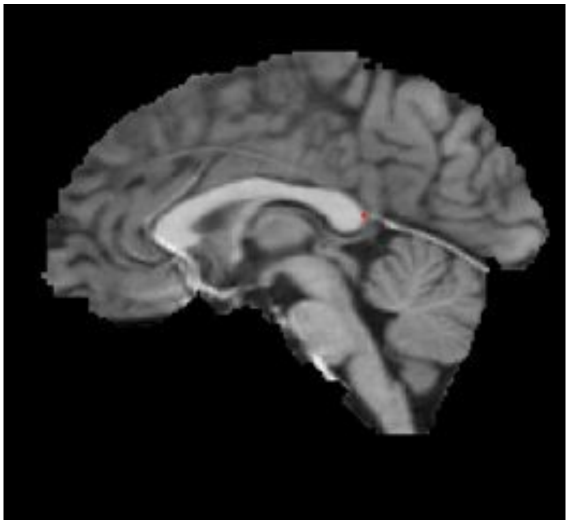

SAGITTAL

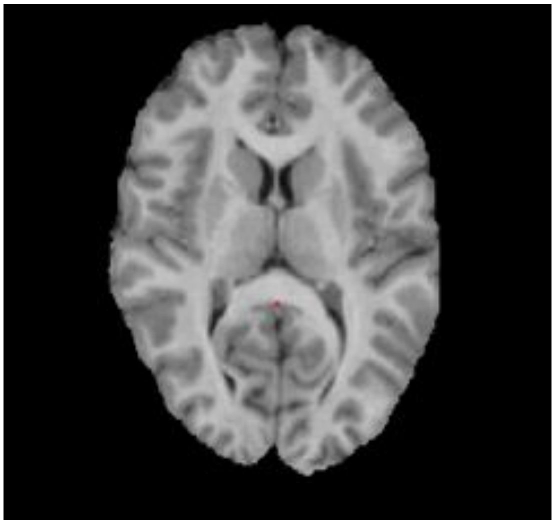

AXIAL

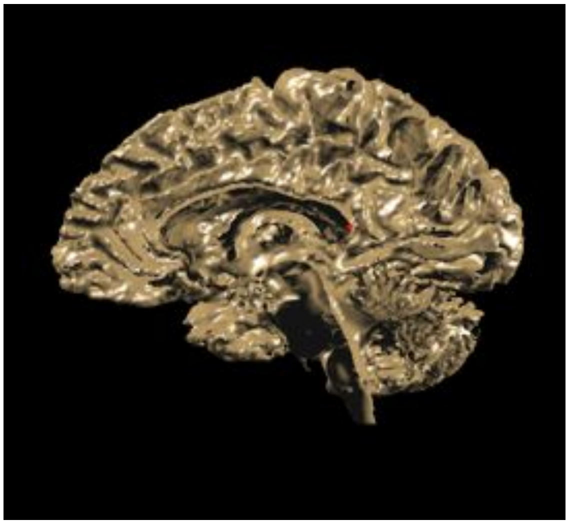

3D

Anterior Commissure (AC)

<u>Definition</u>: The midpoint of the anterior commissure at the midsagittal plane of the diencephalon.

<u>Location</u>: Using the axial view, identify the slice where the anterior commissure has 4 arms coming off a horizontal – two long anterior arms that come off the ends of the horizontal AC and two short posterior arms coming off the middle of the AC. The horizontal AC should be solidly white in color. Place the landmark halfway between the superior and inferior boundaries of the AC and halfway along the AC in the locally-defined mid-sagittal slice.

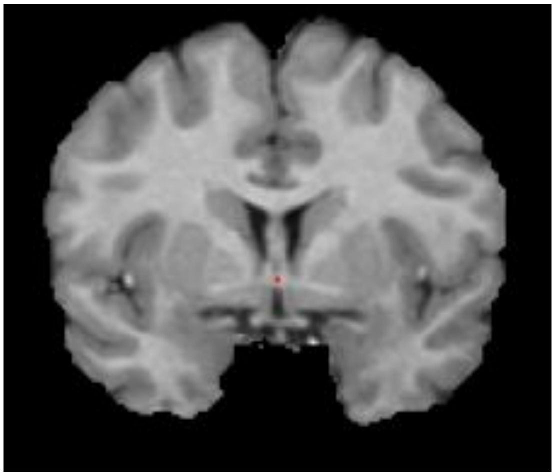

CORONAL

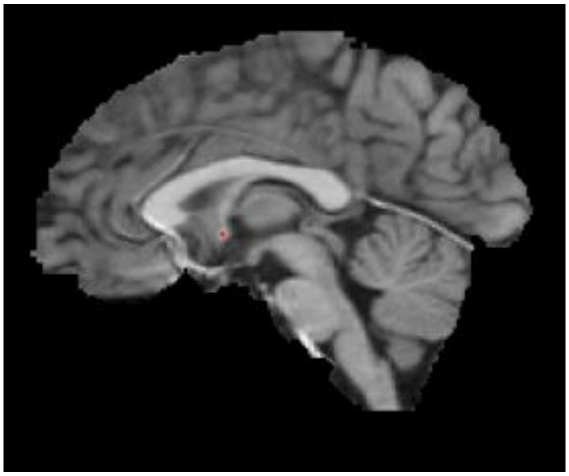

SAGITTAL

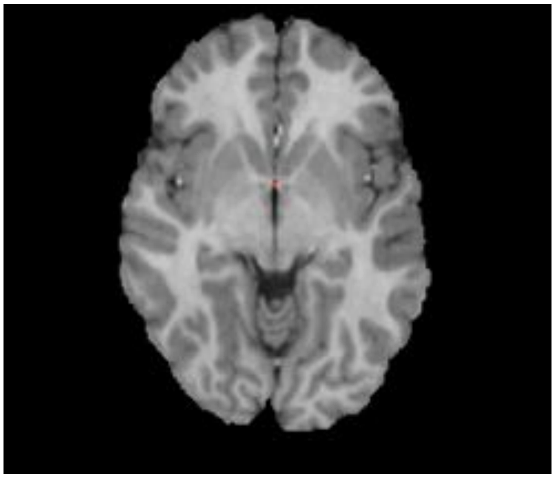

AXIAL

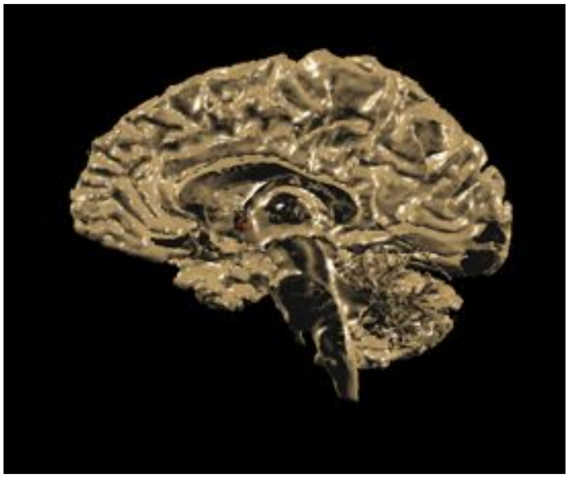

3D

Pons - Inferior (PONSI)

Definition: The inferior-most point of the pons.

Location: Define the midsagittal slice of the inferior pons locally using the coronal and axial views as a guide. Identify the mid-sagittal section of the pons. Using the axial view, scroll from inferior to superior paying attention to the CSF inferior to the pons and anterior to the medulla. Place the landmark on the coronal slice after the pons appears and the CSF becomes encapsulated.

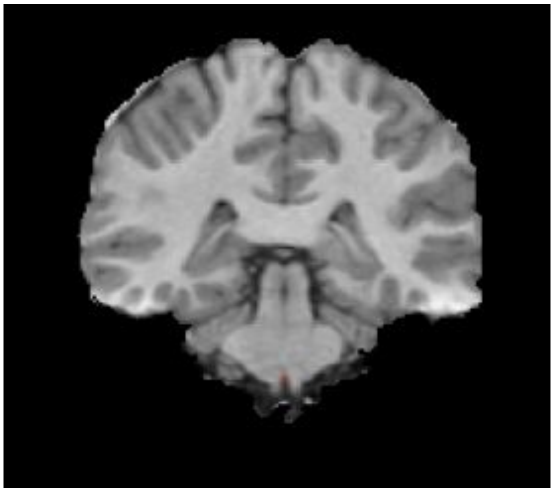

CORONAL

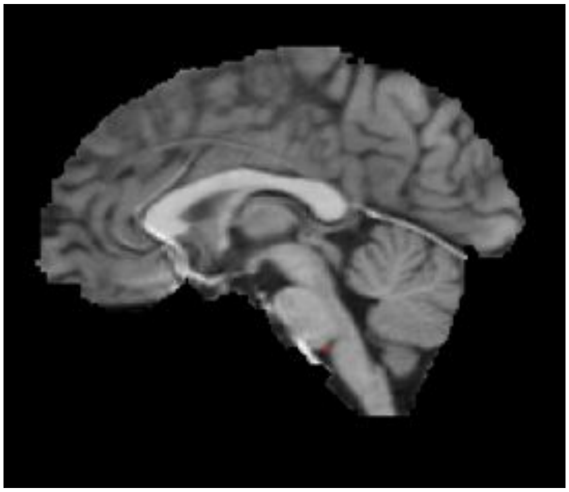

SAGITTAL

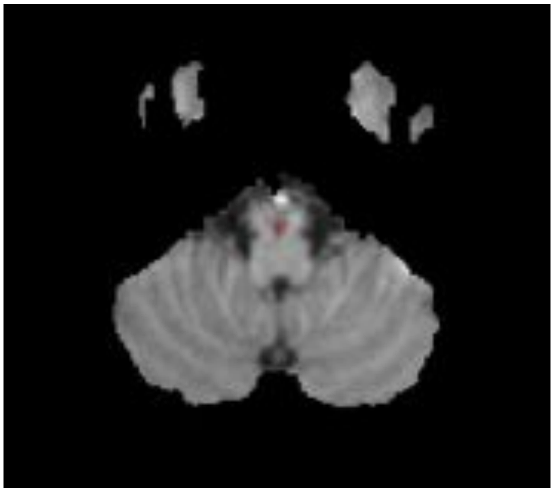

AXIAL

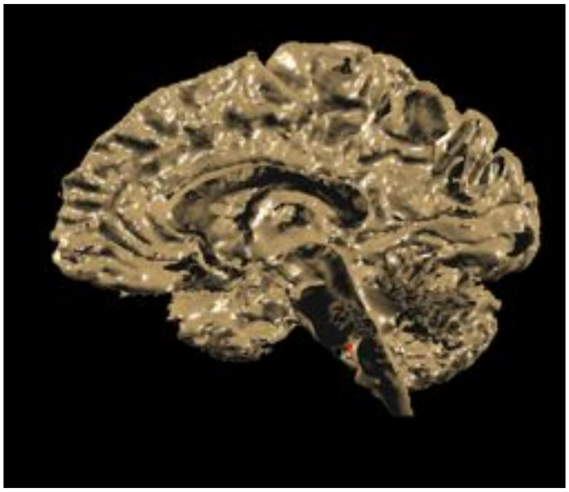

3D

Pons - Superior (PONSS)

Definition: The superior-most point of the pons at the intersection of the pons and the midbrain.

Location: Define the midsagittal slice of the superior pons locally using the coronal and axial views as a guide. Identify the mid-sagittal section of the pons. Using the coronal view, scroll from anterior to posterior paying attention to the CSF superior to the pons and anterior to the midbrain. Place the landmark on the coronal slice after the CSF disappears. (*Note: 'Disappears' is defined here as the slice where only a dark grey circle is visible. One slice anterior, you will see at least one black pixel indicating CSF.)*

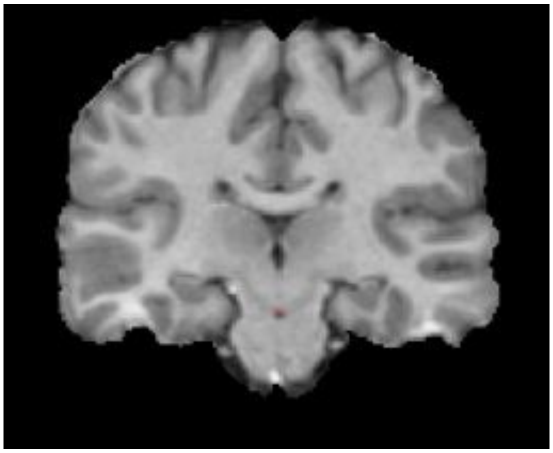

CORONAL

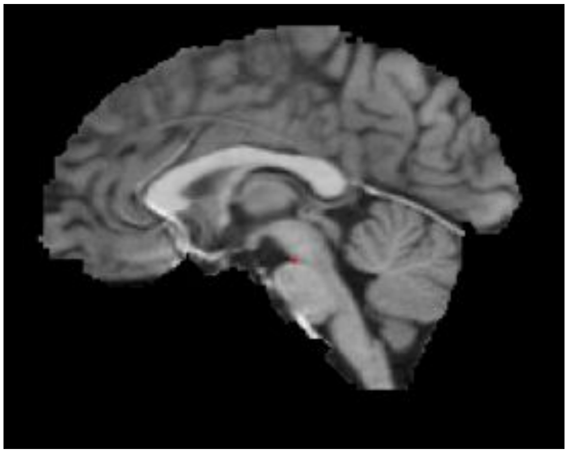

SAGITTAL

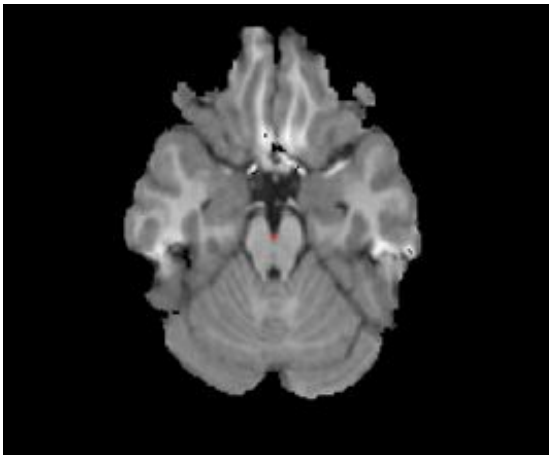

AXIAL

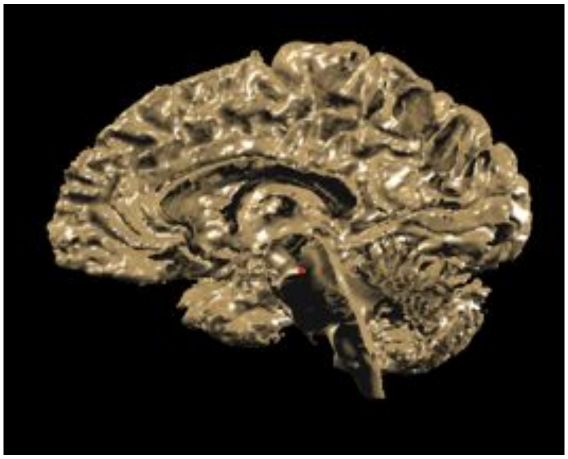

3D

Superior Colliculus (SCOL)

Definition: The centroid of the surface of the superior colliculus.

Location: Use the axial and coronal views to define the mid-sagittal plane of the superior colliculus. Switch to the sagittal view. Imagine a line tangent to the posterior surface of the inferior colliculus and the superior colliculus. Place the landmark at the point where the tangent line hits the superior colliculus.

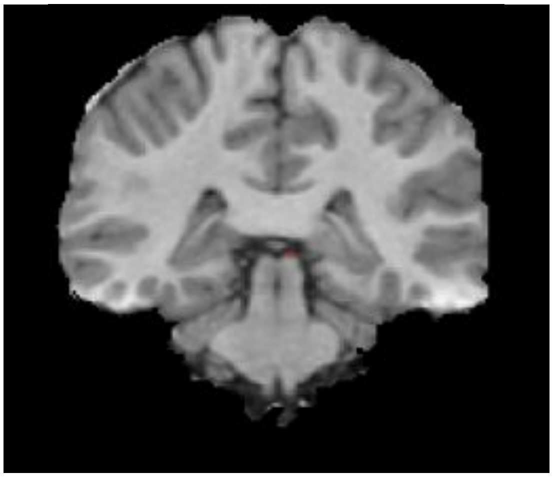

CORONAL

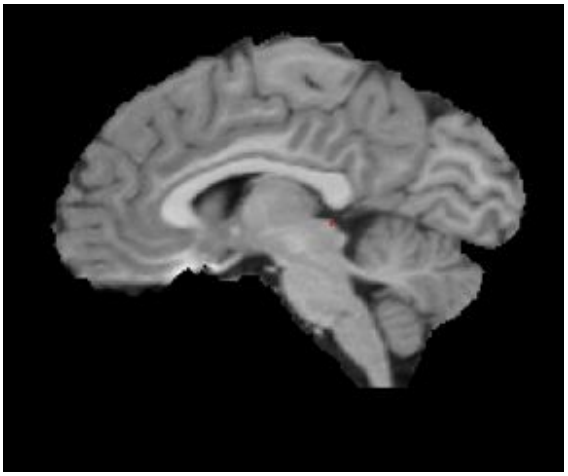

SAGITTAL

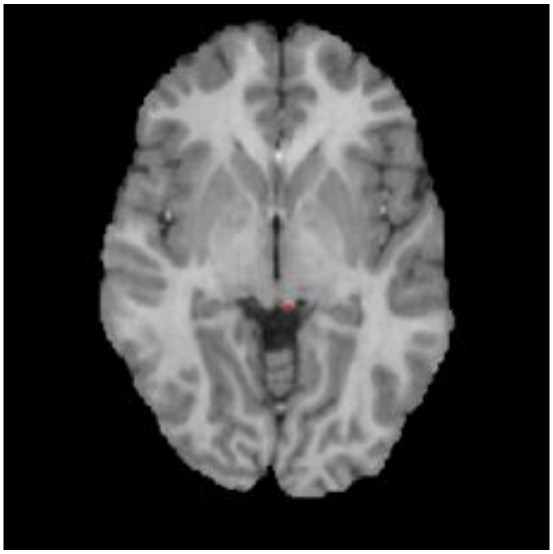

AXIAL

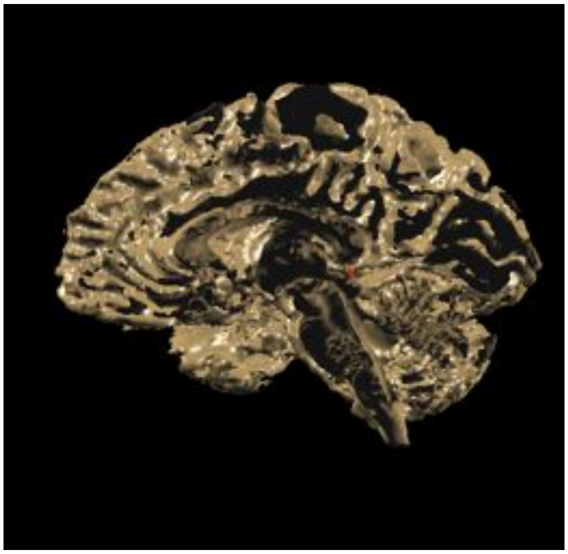

3D

Lateral Ventricle – Anterior Horn (LVA)

<u>Definition</u>: The most anterior point of the anterior horn of the lateral ventricle.

<u>Location</u>: Using the coronal slice, place a landmark on the most anterior point of the anterior horn of the lateral ventricle (the landmark should be placed on the slice with black/dark grey pixels, not the more anterior slice with the light grey pixels). If there is more than one pixel, place the landmark in the center of the CSF. Use the axial view to confirm that it is the most anterior point.

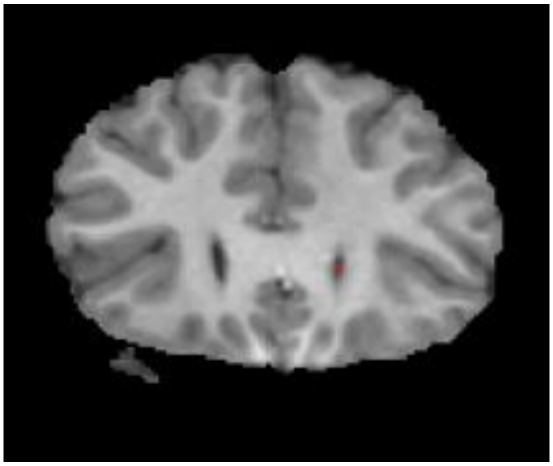

CORONAL

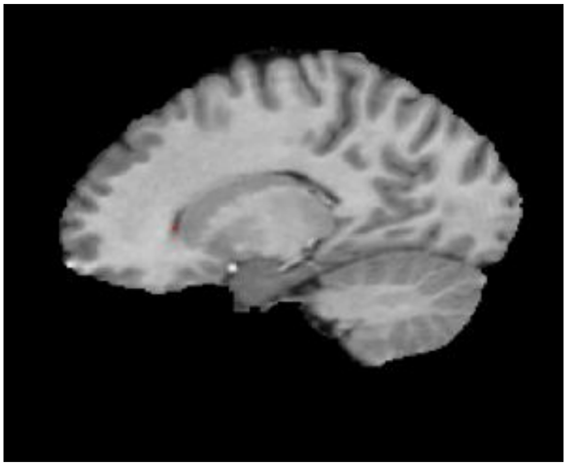

SAGITTAL

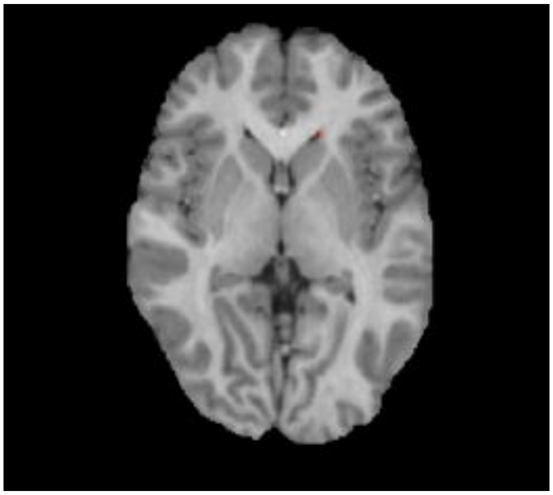

AXIAL

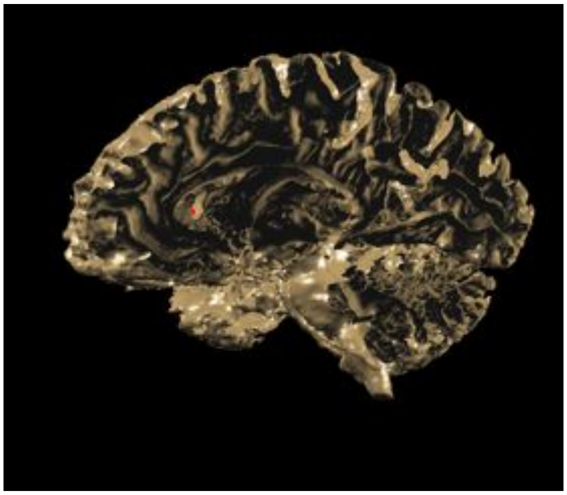

3D

Lateral Ventricle – Inferior Horn (LVI)

Definition: The most inferior point of the inferior horn of the lateral ventricle.

Location: Using the axial view, scroll to the most inferior slice where the inferior horn is visible. If more than one pixel is present, place the landmark in the center of the CSF. Confirm landmark placement using the axial and sagittal views.

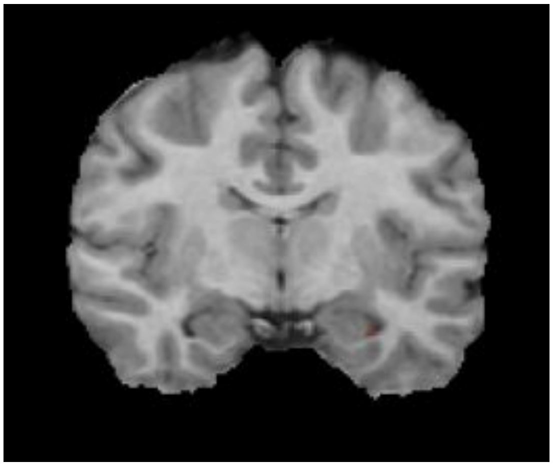
CORONAL

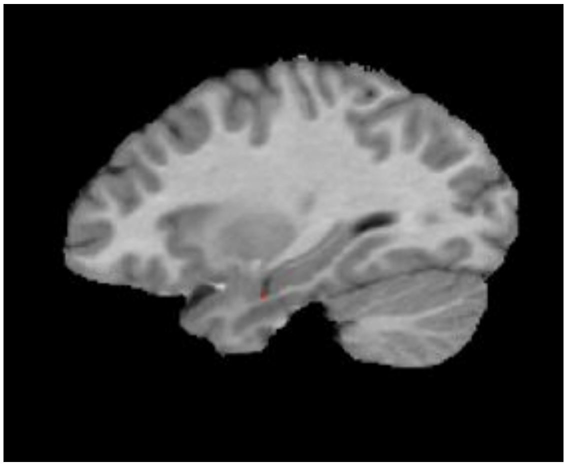
SAGITTAL

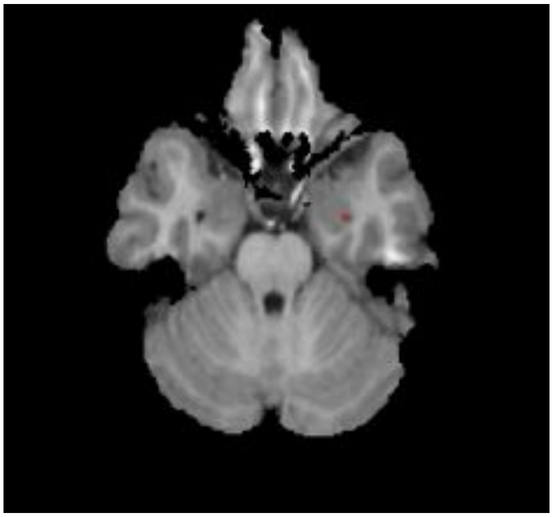
AXIAL

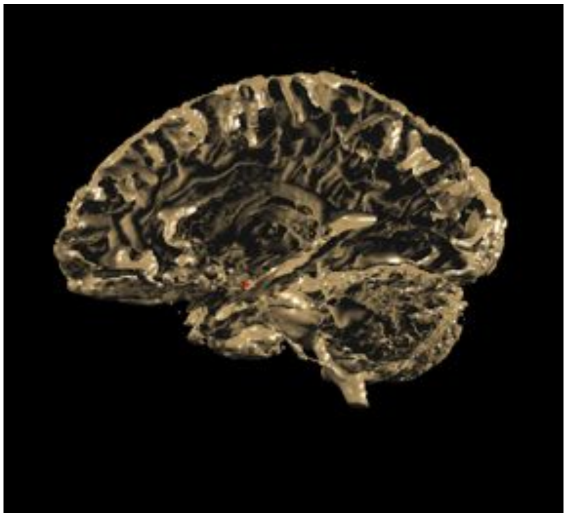
3D

Lateral Ventricle – Posterior Horn (LVP)

Definition: The most posterior point of the posterior horn of the lateral ventricle.

Location: Use the axial view to identify the posterior-most point of the posterior horn. The posterior horn will form a C-shape. In some cases, the ventricles will be compressed (light grey-to-white in color) so the second hook of the C will be hard to identify; do your best to identify the posterior-most point of the posterior horn. In some cases, you really may just be looking for the ghost of an outline.

Example 1:

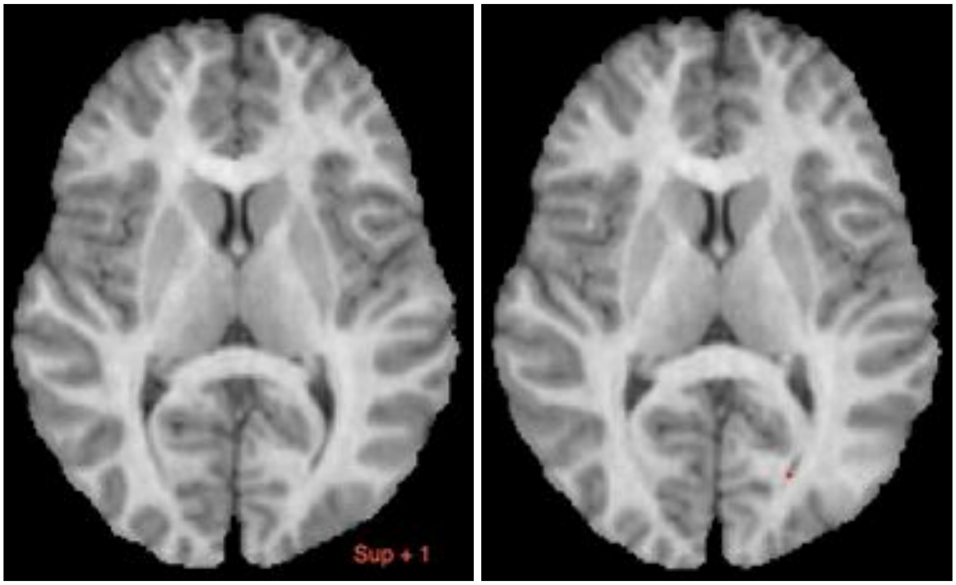

Example 2:

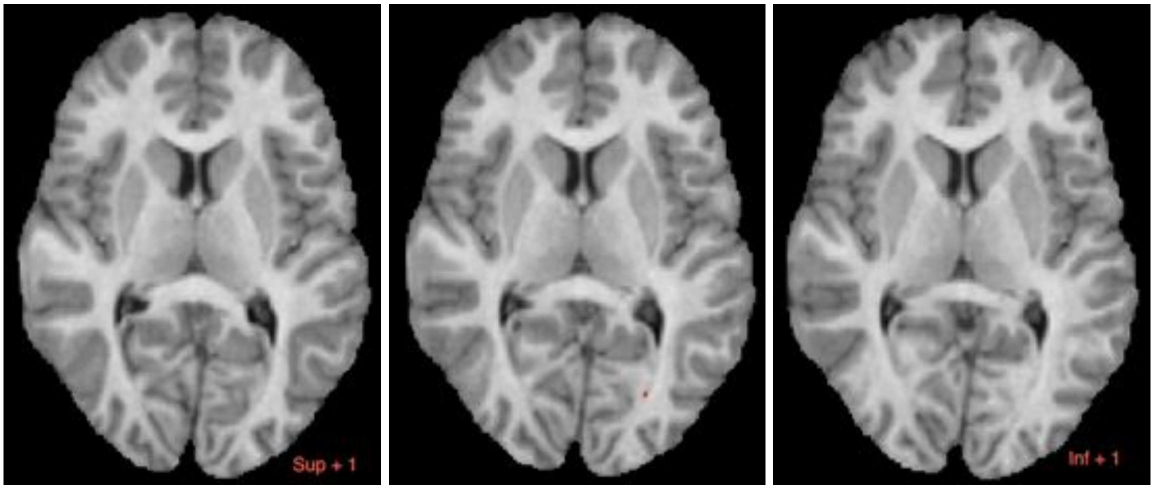

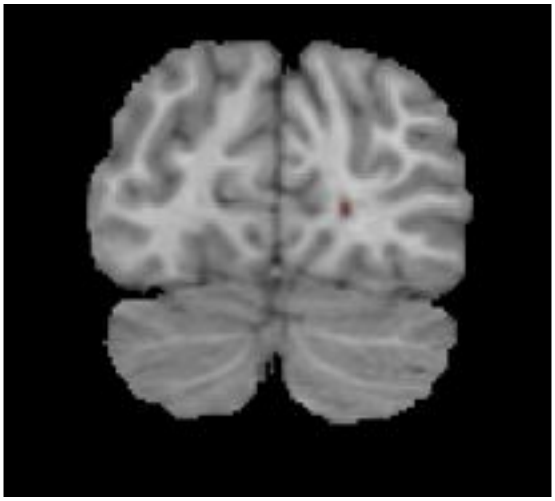

CORONAL

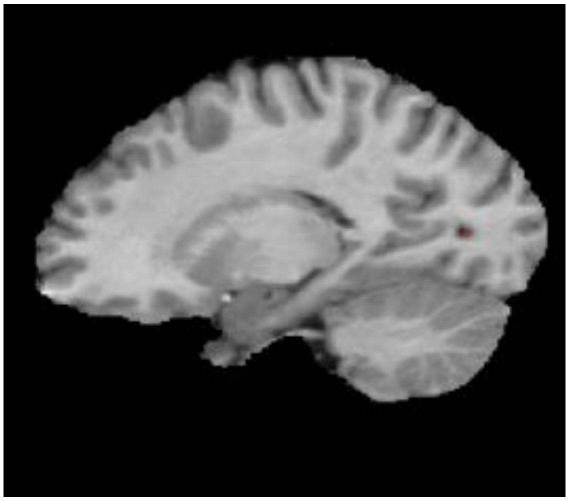

SAGITTAL

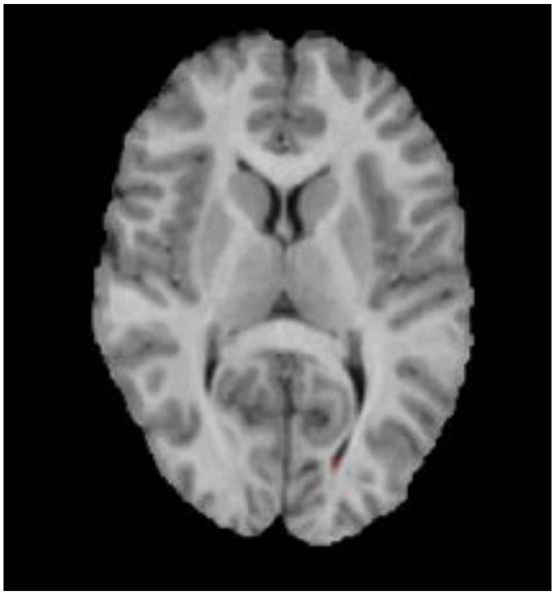

AXIAL

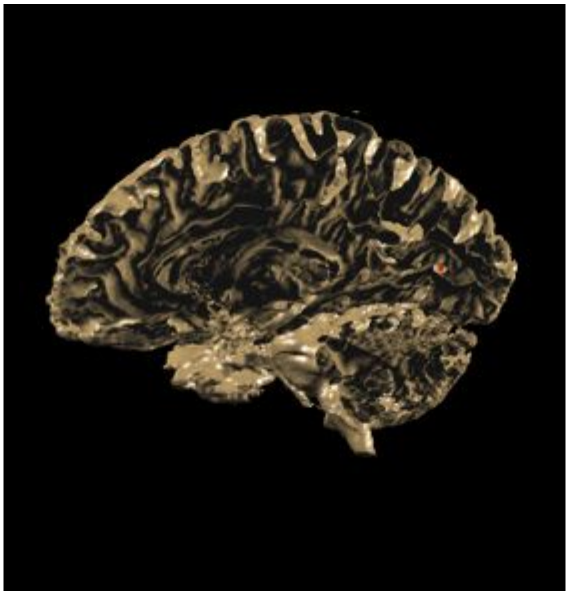

3D
